# Semi-dynamic leach testing of densified silicon-based iodine waste forms

**DOI:** 10.1039/d6ra01079b

**Published:** 2026-04-14

**Authors:** Amanda R. Lawter, Gemma G. Clark, Nancy Escobedo, Jeff Bonnett, Nathan Canfield, Seungrag Choi, Mark E. Bowden, Josef Matyas, R. Matthew Asmussen

**Affiliations:** a Pacific Northwest National Laboratory (PNNL) 902 Battelle Boulevard, P.O. Box 999, MSIN P7-54 Richland WA 99352 USA matthew.asmussen@pnnl.gov +1 (509) 371-7223

## Abstract

A contaminant of concern in nuclear waste, iodine must be immobilized in an iodine waste form (IWF) prior to long-term disposal. A conceptual corrosion release model (CCRM) is required to provide iodine release rates in performance modeling of nuclear waste disposal repositories. To develop a CCRM, corrosion mechanisms of the IWFs and data from consistent test methods are needed to parameterize the model. The present study advances both by assessing the corrosion resistance of IWFs with a series of semi-dynamic leach tests using monolithic IWFs in deionized water (leachant). Experiments were conducted under various test conditions with changes to temperature, leachant replacement, leachant pH, leachant volume, masking, and surface finish to determine if varying these conditions impacted IWF corrosion behaviors. Tests were conducted on two classes of IWFs: (1) iodine-bearing silver-mordenite (AgZ) materials processed by hot isostatic pressing (HIP) at different temperatures, pressures, sizes, and times; and (2) iodine-bearing silver-functionalized silica aerogels (SFA) processed by either HIP or spark plasma sintering (SPS). The corrosion susceptibility of AgZ samples was influenced by HIP temperature and pressure. The SPS SFAs retained iodine far better than HIP SFAs. Additional findings in this study include: (1) the iodine dissolution rate decreased with decreasing temperature, (2) a common ion effect may occur and slow dissolution of the host phase if the leachant is not regularly replaced, (3) pH affects the dissolution rate, and (4) the iodine dissolution rate slows with extended test time (up to 224 days). These parameters should thus be represented when developing a CCRM.

## Abstract 2

Iodine is a radionuclide of concern in nuclear waste repositories. Prior to disposal, iodine must be immobilized in an iodine waste form, the performance of which can be represented in a conceptual corrosion release model.

## Graphical abstract

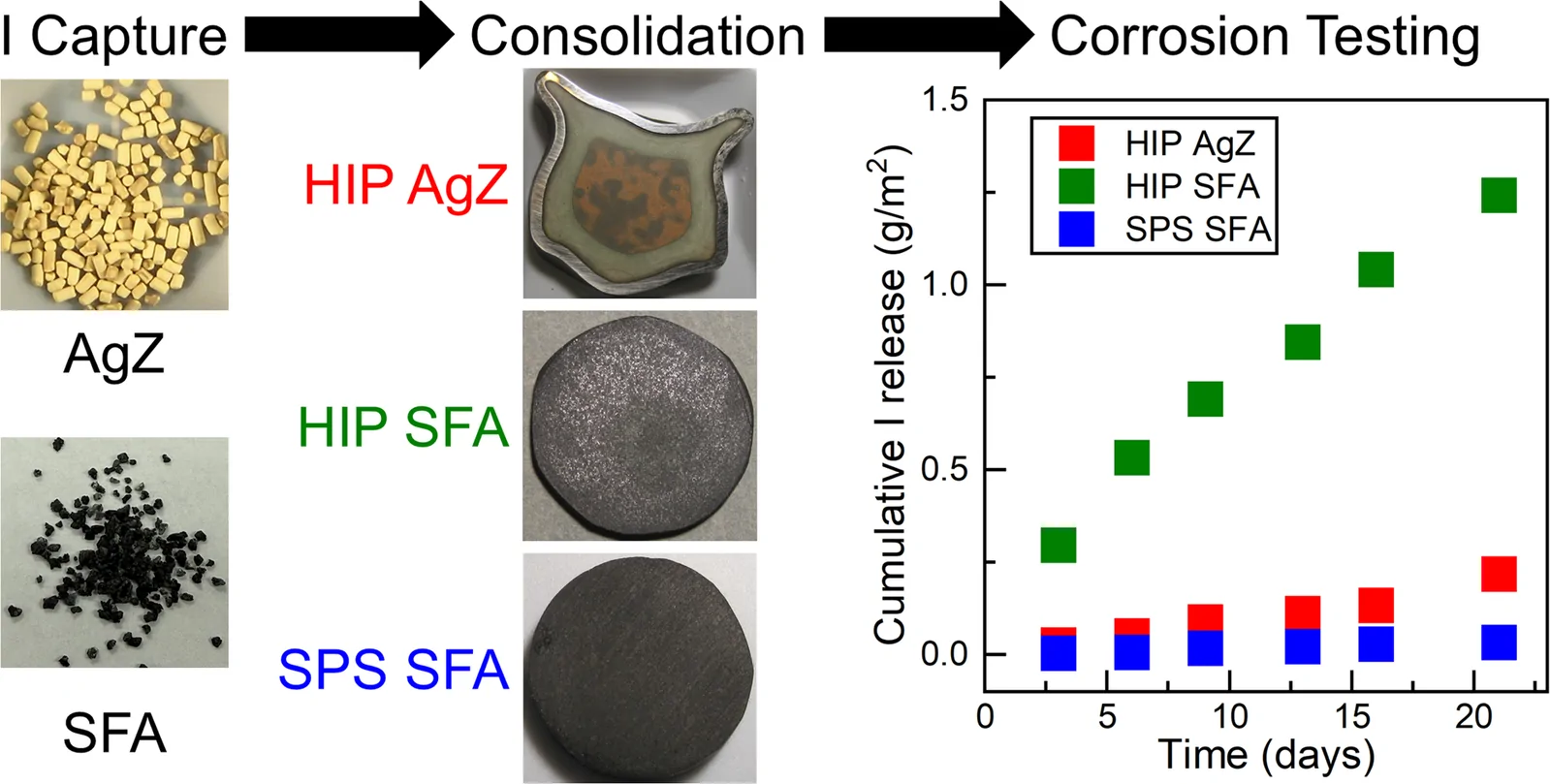

## 1 Introduction

Iodine-129 is a radionuclide of concern in long-term modeling of nuclear waste repositories due to its long half-life (15.7 million years) and high mobility within groundwater as an anion in both oxidized (iodate, IO_3_^−^) and reduced (iodide, I^−^) forms. Iodine is off-gassed during nuclear fuel reprocessing and must be captured to prevent release to the air at levels that exceed regulatory limits of 10 mrem year^−1^.^1^ Any captured iodine must be immobilized in a stable iodine waste form (IWF) for long-term disposal within a repository. Several materials have been produced for iodine capture including zeolites such as mordenite and faujasite,^2–4^ sodalites,^5,6^ apatite,^7,8^ and silver-functionalized silica aerogels (SFA).^9–11^ Following iodine capture, most of these materials require additional processing to be stabilized as a final consolidated IWF. For example, silver mordenite (AgZ) and sodalites can be converted to densified IWFs by hot-isostatic pressing (HIP).^3,5,12^ Other options to generate a consolidated IWF include hot uniaxial pressing,^12^ spark plasma sintering (SPS),^7,10^ vitrification,^13^ and grouting.^14,15^ Further discussion about the various iodine capture technologies can be found in Riley *et al.*,^16^ Asmussen *et al.*,^17^ and Reiser *et al.*^18^

The most common method of iodine capture is chemisorption with a silver (Ag) sorbent. The Ag sorbent generates stable AgI as a sparingly soluble host phase (*K*_sp_ = 8 × 10^−17^). Alternate sorbents would generate different host phases, such as BiI_3_ if Bi was used.^19,20^ While AgI is relatively chemically and thermally stable, individual AgI particles are small and could be transportable as colloids if waste forms are not consolidated. AgI can be chemically destabilized by other chemicals (*e.g.*, sulfide) or redox conditions that may be present in certain disposal scenarios. The stability of AgI-based IWFs can be enhanced by encapsulating the AgI particles within a chemically durable matrix.^21^ Only the AgI host phases exposed to seepage water in a repository would be susceptible to release. Surrounding the host phase with a durable matrix would delay environmental exposure of most of the iodine in the event of a breached canister as the matrix would need to corrode to expose more AgI host phase. The more durable the matrix, the less AgI that would be exposed over time and the less iodine released overall. A durable matrix can be produced by the iodine capture media itself [*e.g.*, hot isostatically pressed (HIPed) AgZ] and enhanced by chemical additions to the iodine capture media during processing (*e.g.*, SiO_2_ additions prior to SPS of SFAs).^22^ The field conditions upon disposal can affect the stability of the host phase and matrix differently, and the stability of both must be addressed in any degradation model.

To assess IWFs for long-term disposal within a repository, a conceptual corrosion release model (CCRM) is required to predict the behavior of the host phase and matrix in controlling iodine release. Any CCRM must be supported with relevant corrosion data of the proposed IWF to parameterize the model. Having data from various IWFs using the same test methodology would allow “head-to-head” comparisons to be made when corrosion testing variables have been isolated and the only differences are those between properties of the samples themselves. Previous corrosion test methods applied to IWFs include ASTM C1662-10 single-pass flow-through test (SPFT),^22^ ASTM C1285-14 product consistency test (PCT),^23^ ASTM C1220-17 [replacement of the Materials Characterization Center-1 (MCC-1) method] static leaching of monolithic waste forms,^24,25^ and ASTM C1308 accelerated test for measuring contaminant releases from solidified waste (a modification of ANSI/ANS 16.1).^5,26,27^ Each of these tests has associated challenges: SPFT and ASTM C1220-17 are labor-intensive, PCT requires destruction of the matrix structure into a fine particle size range (75–150 µm), and ASTM C1308 and the unmodified ANSI/ANS 16.1 have short interval samplings (*e.g.*, 2 hour, 7 hour, and 24 hour samplings) that do not provide information about the long-term behavior of contaminants.^28,29^ The use of various test methods across studies documented in the literature has limited the ability to perform head-to-head comparisons between IWF types as test variables are different and has made it difficult to capture projected dissolution mechanisms in a CCRM, limiting the ability to define a CCRM for IWFs.

This study was designed to compare the corrosion behavior of two waste form types that can be produced from two commonly studied iodine capture materials: AgZ (*i.e.*, IONEX Ag-900) and SFA. The AgZ waste forms were produced in various sizes by HIP processed at different temperatures and pressures. The SFA waste form samples were produced by both HIP and SPS. The corrosion behaviors of the IWFs were compared using various multi-interval, semi-dynamic leach test with varying interval durations. The study addresses the influence of test interval duration on the corrosion behavior of the two IWF types by including extended test intervals of >60 days for the first time. In addition, the impact of various test variables on the corrosion behavior of AgZ and SFA were evaluated (*e.g.*, polishing, silicone masking, temperature, leachant replacement). The approach presented in this study provides further information on the corrosion mechanisms of AgZ-based and SFA-based IWFs and a dataset against which future IWF development can be compared.

## 2 Experimental

### 2.1 Sample preparation

The IWF samples evaluated in this study are listed in Table 1. Replicate samples were prepared if sufficient material was available. The HIPed AgZ samples were produced at Oak Ridge National Laboratory by loading iodine onto the AgZ (IONEX Ag-900, Molecular Products) *via* vapor capture and then sealing the iodine-loaded AgZ in a 304 stainless steel canister.^2,3^ The canisters were then HIPed at various temperatures (525–900 °C), pressures (100–300 MPa), and durations (3 or 12 hours) to consolidate the material, providing a range of processing variables to assess corrosion behavior. Two sizes of HIP AgZ samples were received by Pacific Northwest National Laboratory (PNNL) for testing: a small form (AgZ-SF, 1 cm to 2 cm diameter) and a large form (AgZ-LF, 3 cm to 5 cm diameter). The large form AgZ samples were loaded with two ratios of iodine-to-silver (33% Ag use and 100% Ag use) such that 33% and 100% of the Ag would react I to form AgI. The HIP AgZ samples were received in the steel HIP containers and sectioned radially by using a diamond blade in a low-speed saw with Isomet^®^ cutting oil as a lubricant.

**Table 1:** Processing conditions and surface area (measured with optical microscopy and ImageJ) of the IWF samples used in the present study. Samples with multiple surface area measurements are from different sample replicates

Waste form type	Sample ID	Processing conditions				Surface area (cm^2^)	Sample prepared in	Sample ID in preparation reports^2,3^
		Temperature (°C)	Pressure (MPa)	Time (h)	Notes			
HIP AgZ (small form)	AgZ-SF-2	700	175	3	—	0.955	Bruffey *et al.* (2017)^3^	HIP-17-2
	AgZ-SF-6	900	100	3	—	0.898		HIP-17-6
	AgZ-SF-8	900	175	3	—	0.871		HIP-17-8
	AgZ-SF-17	900	175	12	—	1.030		HIP-17-17
	AgZ-SF-18	525	100	3	—	1.347		HIP-17-18
	AgZ-SF-22	900	175	3	Dried at 450 °C	1.384		HIP-17-22
HIP AgZ (large form)	AgZ-LF-1	900	300	3	100% Ag use^a^	5.20; 4.79	Bruffey and Jubin (2018)^2^	HIP-18-1
	AgZ-LF-2	Replicate of AgZ-LF-1				4.45; 5.33		HIP-18-2
	AgZ-LF-3	900	300	3	33% Ag use^a^	4.74; 4.82		HIP-18-3
	AgZ-LF-4	Replicate of AgZ-LF-3				3.93; 4.70		HIP-18-4
SPS or HIP SFAs	SPS-SFA-1	1200	70	0.5	0% porosity	2.51	In the present study	Not applicable
	SPS-SFA-2	Replicate of SPS-SFA-1				2.04		
	SPS-SFA-3	Replicate of SPS-SFA-1				1.14		
	HIP-SFA-1	1200	207	0.5	8.1% porosity	4.77		
	HIP-SFA-2	Replicate of HIP-SFA-1				4.00		

a The note “100% Ag use” was associated with 134–154 mg I per g of AgZ and saturating the AgZ with I to react all Ag to form AgI. “33% Ag use” was associated with 56–74 mg I per g of AgZ and approximately 33% of Ag reacting with I to form AgI.

Similar to the AgZ samples, the SFA samples were prepared at PNNL with vapor-phase iodine loading of the SFA. The iodine-loaded SFA materials were then consolidated using SPS (1200 °C at 70 MPa for 0.5 hours) or HIP (1200 °C at 207 MPa for 0.5 hours) to compare the durability of IWFs processed with multiple techniques. The SFA samples were prepared as pellets with two flat faces and were not sectioned.

### 2.2 Semi-dynamic leach testing

Unless noted otherwise (*i.e.*, Section 2.3 Alternate test variables), the experimental methods described in this section applied to all leach tests. Leach tests were conducted in 60 mL Savillex vessels at 90 °C using double deionized water, referred to as DDI, >18.0 MΩ cm, meeting Type I standards per ASTM D1193-06(2018), as the leachant. The target surface-area-to-volume (*S*/*V*) ratio of the sample to the leachant was 0.1 cm^−1^ (1 cm^2^ : 10 mL). The HIP AgZ and HIP SFA coupons had one face exposed and the other masked with room-temperature vulcanizing (RTV) silicone or mounted in epoxy resin (Struers Epothin). The samples were placed directly into the test vessel with the single polished side facing up and were completely submerged in the leachant. The primary source of constituent release was the top exposed face. The exposed surface area (one face) of each coupon in the test was imaged with optical microscopy and quantified using ImageJ.^30^ ImageJ was calibrated to a ruler or scale bar in each photo to acquire the ratio of pixels to millimeters. For each sample, the surface area was estimated 3–4 times by manually outlining the perimeter of the sample and taking the average area. The standard deviation of multiple surface area measurements was around 4%. Since the SPS SFA samples did not have a steel wall like the HIP AgZ, the side walls contributed to release and were included in the surface area calculation. IWF samples were polished to 600 grit (30 µm) using silicon carbide sandpaper.

Two control tests were included for each sampling: (1) a DDI control to detect any potential influence of the reactor on the leachate and (2) solutions of DDI spiked with 10 ppm or 100 ppm iodine (from NaI) to determine if any iodine loss occurred due to interactions with the reactor vessel. Both control tests were conducted in the same type of Savillex reactors as the tests with IWF materials and neither showed a measurable impact on the leachate.

At the end of each interval, leachate samples were collected from the reactors and placed into separate 20 mL scintillation vials for analysis. After the leachate aliquot had been collected, the IWF sample was transferred to a clean 60 mL Savillex “rinse” reactor. Each sample was rinsed with DDI in the “rinse” reactor to ensure no carryover of dissolved species to the new leachant. Then the rinse water was discarded. The original reactor was rinsed with DDI and dried with a clean single ply wipe (Kimwipe) prior to transferring the IWF sample back to the reactor. The target volume of DDI, already at 90 °C, was added to the test reactor containing the sample. The mass of water added was recorded to determine the volume added (the density of water at 90 °C of 0.9951 g mL^−1^ (ref. 31) was rounded to 1 g mL^−1^). The reactors and the replacement DDI were kept out of the oven for the shortest duration possible (∼5 minutes). The replacement DDI was periodically set in a 90 °C oven between samplings to keep the water temperature at 90 °C prior to contacting the test sample.

The concentration of species in the leachate were measured with inductively coupled plasma optical emission spectroscopy (ICP-OES, PerkinElmer Optima 8300) for Si (detection limit 26 µg L^−1^) and Ag (detection limit 8.8 µg L^−1^) and inductively coupled plasma mass spectroscopy (ICP-MS, either PerkinElmer ELAN DRC-II or ThermoFisher X-Series II) for I-127 (detection limit 0.0073 µg L^−1^, prior to dilution). The normalized dissolution rate (NDR) with respect to a specific species (*X*) was determined using eqn (1):1
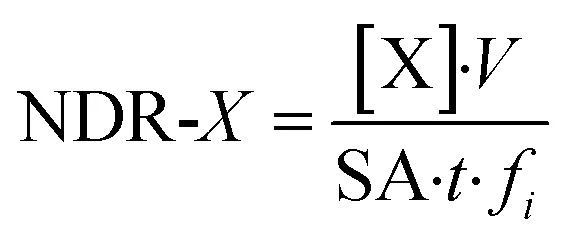
where NDR-*X* is the normalized dissolution rate of species *X* in g m^−2^ d^−1^, [X] is the measured concentration of the species in g L^−1^, *V* is the leachant volume in L, SA is the sample surface area exposed to leachant in m^2^, *t* is the interval time in *d*, and *f*_*i*_ is the measured mass fraction of the element in the sample. The polished top surface area was used for the HIP AgZ sample, as a steel wall protected the sides and the downward face on the reactor floor was masked with silicone. On the SFA samples, the side walls were included in the surface area calculation. A cumulative normalized mass loss (CNL) was calculated from the corrosion data by summing the mass of the constituent (*X*) released at each time interval to obtain the cumulative mass lost, then normalizing by the weight (wt)% of the element within the sample. This is given in eqn 22
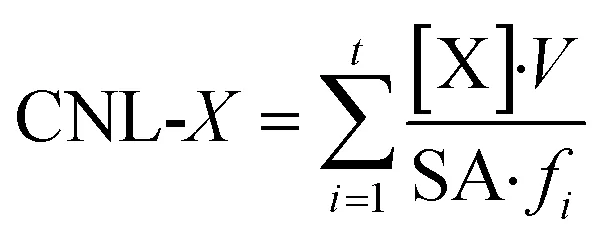
where CNL-*X* is in g m^−2^ at time *t* (days) for species *X*, [X] is the measured concentration of the species in g L^−1^, *V* is the leachant volume in L, *SA* is the sample surface area in m^2^, and *f*_*i*_ is the measured mass fraction of the element in the sample.

### 2.3 Alternate test variables

To assess the sensitivity of the IWF, various test conditions were altered including sampling interval, temperature, leachant replacement, leachant pH, *S*/*V* ratio, masking, and surface finish (degree of polish). A summary of the test conditions used for each sample is shown in Table 2. Efforts were made to limit corrosion damage during testing to allow for repeat use of the samples in subsequent tests. However, in some cases, replicate tests were not possible due to extensive corrosion and sample loss. Only one test variable was altered at a time. Samples showing altered test variables for multiple variables in Table 2 were reused or replicate samples were used for separate tests involving different altered test variables. When reusing samples, the sample was re-polished between tests.

**Table 2:** Experimental matrix for tests performed on various IWFs

Waste form type	Sample ID	Sampling schedule^a^ (Sec 2.3.1)	Test temperature (Sec 2.3.2)	Leachant replacement (Sec 2.3.3)	Leachant pH (Sec 2.3.4)	*S*/*V* ratio (Sec 2.3.5)	Masking^b^ (Sec 2.3.6)	Surface finish (Sec 2.3.7)
HIP AgZ (small form)	AgZ-SF-2	A	90 °C	Yes	Unbuffered DDI	0.1 cm^−1^	Yes	600 grit
	AgZ-SF-6	A	90 °C	Yes	Unbuffered DDI	0.1 cm^−1^	Yes	600 grit
	AgZ-SF-8	A, B	23 °C, 40 °C, 90 °C	Yes	Unbuffered DDI	0.1 cm^−1^	Yes	600 grit
	AgZ-SF-17	B	23 °C, 40 °C, 90 °C	Yes	Unbuffered DDI	0.1 cm^−1^	Yes	600 grit
	AgZ-SF-18	A	90 °C	Yes	Unbuffered DDI	0.1 cm^−1^	Yes	600 grit
	AgZ-SF-22	B	23 °C, 40 °C, 90 °C	Yes	Unbuffered DDI	0.1 cm^−1^	Yes	600 grit
HIP AgZ (large form)	AgZ-LF-1	A, C	90 °C	Yes	4, 7, 11	0.1 cm^−1^	Yes	600 grit, 1200 grit
	AgZ-LF-2	A, B, D	90 °C	Yes	Unbuffered DDI	0.1 cm^−1^, 0.01 cm^−1^	Yes	2400 grit, Coll^c^
	AgZ-LF-3	A, B, D	90 °C	Yes, no	Unbuffered DDI	0.1 cm^−1^	Yes	600 grit, 1200 grit
	AgZ-LF-4	A, B, C	90 °C	Yes	4, 7, 11	0.1 cm^−1^, 0.01 cm^−1^	Yes	600 grit, 2400 grit, Coll^c^
SPS or HIP SFAs	SPS-SFA-1	A, B	90 °C	Yes	Unbuffered DDI	0.1 cm^−1^	No, yes	600 grit
	SPS-SFA-2	A, B	90 °C	Yes	Unbuffered DDI	0.1 cm^−1^, 0.3 cm^−1^	No, yes	600 grit
	SPS-SFA-3	C	90 °C	Yes	4, 7, 11	0.1 cm^−1^	No	600 grit
	HIP-SFA-1	A	90 °C	Yes	Unbuffered DDI	0.1 cm^−1^	No, yes	600 grit
	HIP-SFA-2	A, B	90 °C	Yes	Unbuffered DDI	0.1 cm^−1^, 0.8 cm^−1^	No, yes	600 grit

a Sampling schedules performed with Interval A, Interval B, Interval C, and Interval D abbreviated “A,” “B,” “C,” and “D”.

b Masking with room-temperature vulcanizing (RTV) silicone or mounted in epoxy resin.

c Polished with colloidal silica.

#### 2.3.1 Sampling interval

Many test methods exist for the evaluation of monolithic or powdered waste forms in semi-dynamic leach tests, each with a unique sampling schedule and targeted mechanisms for observation. This creates a challenge for head-to-head comparisons if data comes from tests with different variables or discrepancies in corrosion rates measured with different tests when used in a CCRM. A comparison of the leaching intervals of common test methods is given in the SI, Table S1.

In the present study, four different testing intervals were considered as are shown in Table 3. The first, Interval A, used cumulative sampling intervals of 1, 2, 3, 7, 11, 15, and 21 days. An alternate testing schedule (Interval B) was used to compare the effects of different sampling intervals on HIP AgZ corrosion behavior (3, 6, 9, 13, 16, and 22 days). Samples tested with Interval B were evaluated for the feasibility of using a single-test interval as a screening test to acquire trends between samples. Additional sampling schedules (Interval C and Interval D) were applied to a selection of large form HIP AgZ samples and one SPS SFA sample while testing the effects of leachant pH.

**Table 3:** Summary of semi-dynamic leach testing intervals

Test method	Sampling schedule, cumulative time (days)
Interval A	1, 2, 3, 7, 11, 15, 21
Interval B	3, 6, 9, 12, 16, 21
Interval C	1, 2, 3, 4, 7
Interval D	1, 2, 3, 4, 7, 14, 28, 56, 112, 224

#### 2.3.2 Temperature

Waste form dissolution measurements are commonly performed at elevated temperatures to accelerate dissolution. Analysis is then done with an expected Arrhenius response to temperature increases assuming the dissolution mechanism is not altered with temperature. This behavior was evaluated for the IWFs by performing tests at 23 °C, 40 °C, and 90 °C using the small-form HIP AgZ samples (AgZ-SF-8, AgZ-SF-17, and AgZ-SF-22).

#### 2.3.3 Leachant replacement

To investigate the influence of leachant refreshment on HIP AgZ corrosion, one test set was performed on a HIP AgZ (AgZ-LF-3) in which the leachant was not replaced during the test. Instead, a 250 µL aliquot was taken for each sampling event and the HIP AgZ coupon was neither rinsed nor placed in a reactor with fresh leachant at each interval. The leachant volume was approximately 48 mL (corresponding to the AgZ-LF-3 surface area of ∼4.8 cm^2^), so collecting 250 µL aliquots resulted in negligible (0.5%) leachant removal. The cumulative sample intervals followed Interval A (1, 2, 3, 7, 11, 15, and 21 days). The NDR was calculated from the change in concentration between each interval.

#### 2.3.4 Leachant pH

IWFs may be exposed to a range of environmental conditions depending on the repository. Considering the impact of the water chemistry of the disposal site, leach tests were performed under different pH conditions. Instead of DDI water, pH buffers at pH 4 (Fisher), pH 7 (Fisher), and pH 11 (Ricca) served as the leachant. Tests were performed at 90 °C or 40 °C. Sample schedules followed Interval C (1, 2, 3, 4, and 7 days) or Interval D (1, 2, 3, 4, 7, 14, 28, 56, 112, and 224 days). Leach tests with the pH buffers were performed on the large form HIP AgZ samples (AgZ-LF-1, AgZ-LF-2, AgZ-LF-3, AgZ-LF-4) and on one SFA sample (SPS-SFA-3).

#### 2.3.5 Surface-area-to-volume ratio

Within a repository, the disposed waste forms will experience an evolving near-field environment. Depending on water migration rates and the repository type, the near field will most likely become increasingly saturated with dissolved components from the waste forms. For silicon-based materials, a common ion effect could arise from this behavior and the corrosion rate of the material would slow with increased dissolved component concentrations in the near field.^32^ Differences in the near-field environment were represented in the semi-dynamic leach tests of the present study by changing the *S*/*V* ratio from 0.1 cm^−1^ used in most tests to *S*/*V* ratios of 0.01, 0.3, and 0.8 cm^−1^. Smaller ratios represent test conditions that are more diluted, which may result in faster corrosion of the waste form. These results will help determine if corrosion release models need to account for the degree of saturation in the field. Tests at lower *S*/*V* were performed in 500 mL high-density polyethylene bottles instead of 60 mL Savillex vessels because a larger container was needed to hold larger leachant volumes.

#### 2.3.6 Masking

To preserve the IWF samples for repetitive measurements, corrosion was limited to a single face for most tests. In previous development efforts,^33^ a single face of the IWF was exposed and the remaining faces were masked with RTV silicone. An increased release of iodine and potassium was observed in some of those tests and correlated to silicone masks that were not fully cured at the time of the test, thus leading to an artificially high NDR-I. In the present study, the SPS-SFA-1, SPS-SFA-2, HIP-SFA-1, and HIP-SFA-2 samples were used to assess if the bottom face of the sample can be left unmasked and placed downward on the reactor floor, lessening its exposure to solution and removing possible risk of contamination from silicone masking. The corrosion rates of samples with and without a mask protecting the bottom face of the sample were compared.

#### 2.3.7 Surface finish

Following the initial sectioning (if applicable), or in preparation for repeat test runs, the target face of the IWF samples was polished to ensure a fresh surface was exposed in the new test. Samples were polished to varying degrees of fineness to assess the impact of surface finish on corrosion. HIP large form samples (AgZ-LF-1, AgZ-LF-2, AgZ-LF-3, AgZ-LF-4) were polished with colloidal silica (Coll), 2400 grit silicon carbide (SiC), 1200 grit SiC, or the standard 600 grit SiC.

### 2.4 Microscopy

Sample surfaces were imaged using scanning electron microscopy (SEM) with a JSM-7001F microscope (JEOL USA, Inc.). The elemental distribution and compositions were determined using energy dispersive X-ray spectroscopy (EDS) with an XFlash 6|60 EDS Si-drift detector (Bruker) for elemental mapping and spot analysis. Element concentrations determined by EDS were used for composition measurements due to the heterogeneity of sample microstructures, unless otherwise stated. An average from multiple spots (3–5) at 250× magnifications (∼500 µm × ∼500 µm) was used for the compositions.

### 2.5 Micro-X-ray diffraction (µXRD)

X-ray diffraction (XRD) patterns were collected from selected regions of the specimens using a Rigaku D-Max Rapid II microbeam diffractometer. X-rays were generated from a rotating Cr anode source (*λ* = 2.2910 Å) operated at 35 kV and 25 mA and focused to give a beam approximately 300 µm in diameter. The beam was positioned on areas having different appearances by using a micrometer XY stage and optical camera integral to the diffractometer. The diffracted intensities were recorded on a large two-dimensional image plate during a 10 min exposure and integrated to give a one-dimensional powder diffraction profile. Crystalline compounds were identified by comparing sample patterns to reference patterns (International Centre for Diffraction Data, PA) using JADE software (Materials Data Incorporated, CA). XRD patterns collected before consolidation (HIP or SPS) are not shown in the present study but have been published previously, both before and after iodine loading.^34,35^

## 3 Results and discussion

### 3.1 Pre-corrosion sample characteristics

**Fig. 1:**
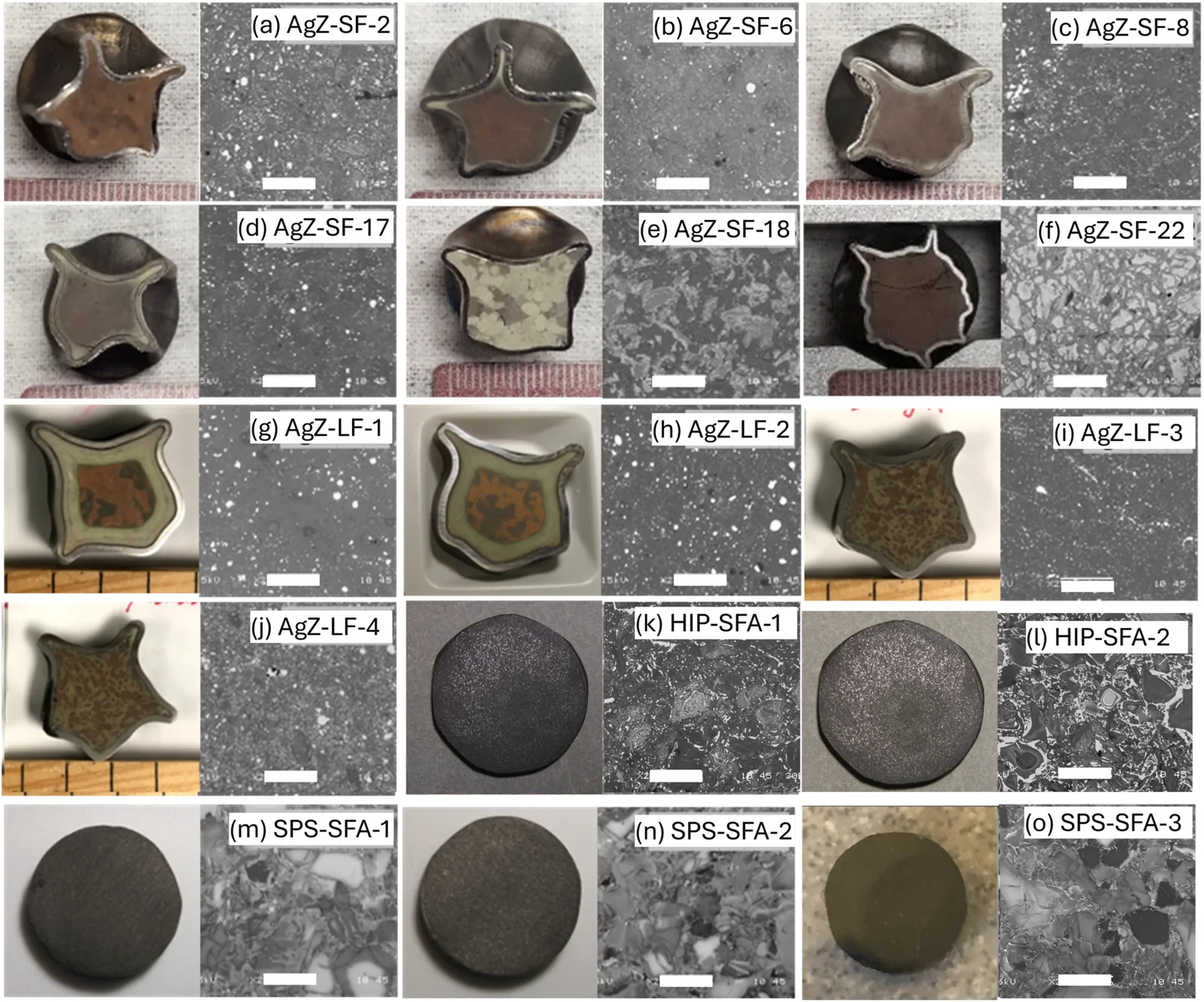
Photographs and SEM micrographs of (a) AgZ-SF-2, (b) AgZ-SF-6, (c) AgZ-SF-8, (d) AgZ-SF-17, (e) AgZ-SF-18, (f) AgZ-SF-22, (g) AgZ-LF-1, (h) AgZ-LF-2, (i) AgZ-LF-3, (j) AgZ-LF-4, (k) HIP-SFA-1, (l) HIP-SFA-2, (m) SPS-SFA-1, (n) SPS-SFA-2, (o) SPS-SFA-3 samples prior to leach testing. All samples were imaged with SEM at the same magnification prior to leach testing (all scale bars for SEM images are represented by white bars and are 100 µm).

Photographs of the IWF samples used in this study and the SEM micrographs of their microstructures are shown in Fig. 1. Samples contained various compositions within the microstructures. The measured surface compositions of the samples are provided in the SI (Table S2). The composition values were adjusted to remove the carbon contribution that was due to the carbon coating used in the SEM imaging as well as carbon contamination in the SEM. The NDR values were calculated using iodine concentrations measured with EDS. The extent to which iodine volatilized during IWF processing or during EDS analyses is unknown but assumed to be constant in the samples as identical rastering times were used for all SEM-EDS analyses. The IWF compositions based on the nominal iodine concentration prior to processing differed from the EDS values by 15–20% in the SFA samples and 0–9% in the AgZ-LF samples. The resulting NDRs for the SFA and AgZ-LF samples using the nominal values are presented in the SI (Tables S3, S4 and Fig. S1, S2).

EDS elemental maps of the AgZ-SF samples have been reported previously in detail^22^ and for the samples in this study are presented in the SI (Fig. S3–S7). The microstructure was a mix of Si–Al rich matrix phases with varying alkali content (*e.g.*, K, Mg) and the AgI host phases of different sizes dispersed in the space between these Si–Al-rich phases. The AgZ-SF samples were prepared under various processing conditions (HIP temperature, pressure, time, and water content), which altered the sample microstructure. In general, lower-temperature HIP samples (AgZ-SF-2 and AgZ-SF-18) generated larger phase domains with more isolations of Na, K, Si, and Al. Increasing HIP pressure and HIP time decreased the size of the host phase isolations and increased the size of alkali-rich regions. Drying of the AgZ-SF-22 sample prior to HIP produced a microstructure with sharper grain edges.

The difference between the two types of AgZ-LF samples was the overall Ag utilization through loading with iodine (100% Ag use: 134–154 mg I per g AgZ; 33% Ag use: 56–74 mg I per g AgZ). However, the two AgZ-LF sample types had dramatically different visual microstructures. The replicate AgZ-LF-1 and AgZ-LF-2 samples had 100% Ag use and were composed of a brown core with a camouflage-like pattern surrounded by a lighter green colored region (Fig. 1g and h). The replicate AgZ-LF-3 and AgZ-LF-4 samples, which had a 33% use of Ag, had a randomized camouflage pattern of small brown isolations dispersed between lighter green colored sections (Fig. 1i and j). Further micrographs, corresponding EDS maps and analyses can be found in the SI for the AgZ-LF samples (Fig. S8 and S9) and the HIP and SPS SFA samples (Fig. S10). XRD patterns of AgZ-LF-2 (100% Ag use) and AgZ-LF-3 (33% Ag use) from before and after leach testing, and XRD phases of HIP SFA and SPS SFA are also available in the SI (Fig. S11–S14 and Table S5).

### 3.2 Iodine waste form corrosion testing

The NDR-I provided an indication of the corrosion resistance of the host phase. NDR-Ag provided some information about the corrosion resistance of the host phase but to a lesser extent because not all Ag^+^ may remain in solution and not all Ag in the system is AgI. Additionally, silver can precipitate (governed by solubility or redox conditions) while iodine does not. This could explain why the silver-to-iodine molar ratio was not 1 : 1 in most leach tests. As such, the NDR-I (not NDR-Ag) should be used when modeling host phase corrosion and waste form performance. The NDR-Si provided a measure of the corrosion resistance of the microstructure matrix.

#### 3.2.1 Interval A sampling schedule

**Fig. 2:**
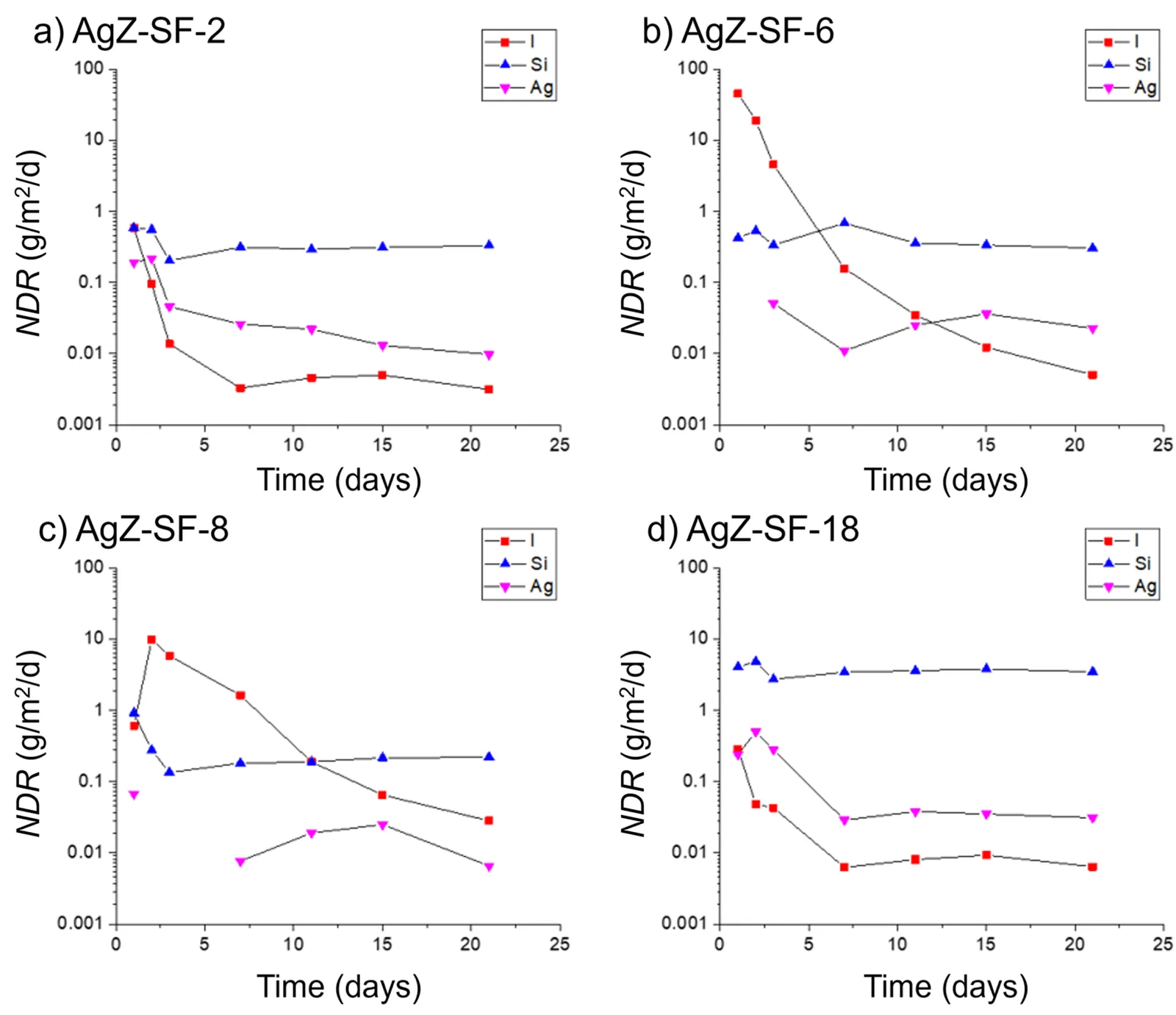
The NDR of iodine (red squares), silicon (blue upward triangle), and silver (pink downward triangle) measured at 90 °C for (a) AgZ-SF-2 (HIPed at 700 °C and 175 MPa), (b) AgZ-SF-6 (HIPed at 900 °C and 100 MPa), (c) AgZ-SF-8 (HIPed at 900 °C and 175 MPa), and (d) AgZ-SF-18 (HIPed at 525 °C and 100 MPa).

Interval A semi-dynamic leach tests followed a leachant exchange schedule of 1, 2, 3, 7, 11, 15, and 21 days (cumulative). The NDRs based on I (NDR-I), Ag (NDR-Ag), and Si (NDR-Si) were calculated for AgZ-SF-2, AgZ-SF-6, AgZ-SF-8, and AgZ-SF-18 (Fig. 2). Unfortunately, replicate tests were unable to be performed due to lack of sample material. Only one processing condition (temperature or pressure) was different amongst the samples, allowing for a direct comparison.

The small form AgZ samples displayed incongruent dissolution and different corrosion behavior resulting from their processing conditions, as was previously observed in SPFT testing for a similar type of IWF.^22^ All samples had a decreasing NDR-I with time. This observation was likely due to corrosion of the host phase that was initially exposed to the leachant followed by lower host phase exposure until significant matrix corrosion occurred. The lowest NDR-I was observed in AgZ-SF-2 (0.004 g m^−2^ d^−1^ after 7 days), with AgZ-SF-18 showing similar behavior. Both AgZ-SF-6 and AgZ-SF-8 had high initial NDR-I values before decreasing. The AgZ-SF-6 and AgZ-SF-8 were HIPed at higher temperature (900 °C) than the other two samples (AgZ-SF-2 and AgZ-SF-18, HIPed at 700 °C and 525 °C, respectively), suggesting that high HIP temperatures may destabilize some AgI host phase within the waste form or produce some free I that can be readily released. This result aligns with previous testing of similar samples using PCT that showed overall sample durability decreased with HIP temperatures above 900 °C.^36^ Differences in HIP processing conditions also could have induced a crystalline phase transformation of AgI, which could affect the dissolution rate between the different phases.^37^ Using XRD, β-phase and γ-phase AgI were quantified in HIPed SFA samples (Table S5), but unfortunately, it is unknown if any phase transformations occurred during the HIPing, what impact the processing conditions had on the ratio of generation of these phases within different materials, or the difference in actual dissolution rates from the samples. All of these variables were not a focus of the present study but should be a subject of future research.

The NDR-Ag measurements were all between 0.01 g m^−2^ d^−1^ and 0.2 g m^−2^ d^−1^; however, Ag solubility within the leachant may have influenced the NDR-Ag. The matrix dissolution results for the samples HIPed at or above 700 °C (AgZ-SF-2, AgZ-SF-6, and AgZ-SF-8) were similar with NDR-Si measuring between 0.1 g m^−2^ d^−1^ and 1 g m^−2^ d^−1^. However, the AgZ-SF-18 sample HIPed at 525 °C had an NDR-Si of 30 g m^−2^ d^−1^, indicating that the low HIP temperature did not generate a corrosion-resistant matrix to protect the AgI host phase. With time, it can be expected that this sample would continually corrode its matrix, revealing more AgI host phase and leading to increased NDR-I despite the initially low NDR-I for AgZ-SF-18.

**Fig. 3:**
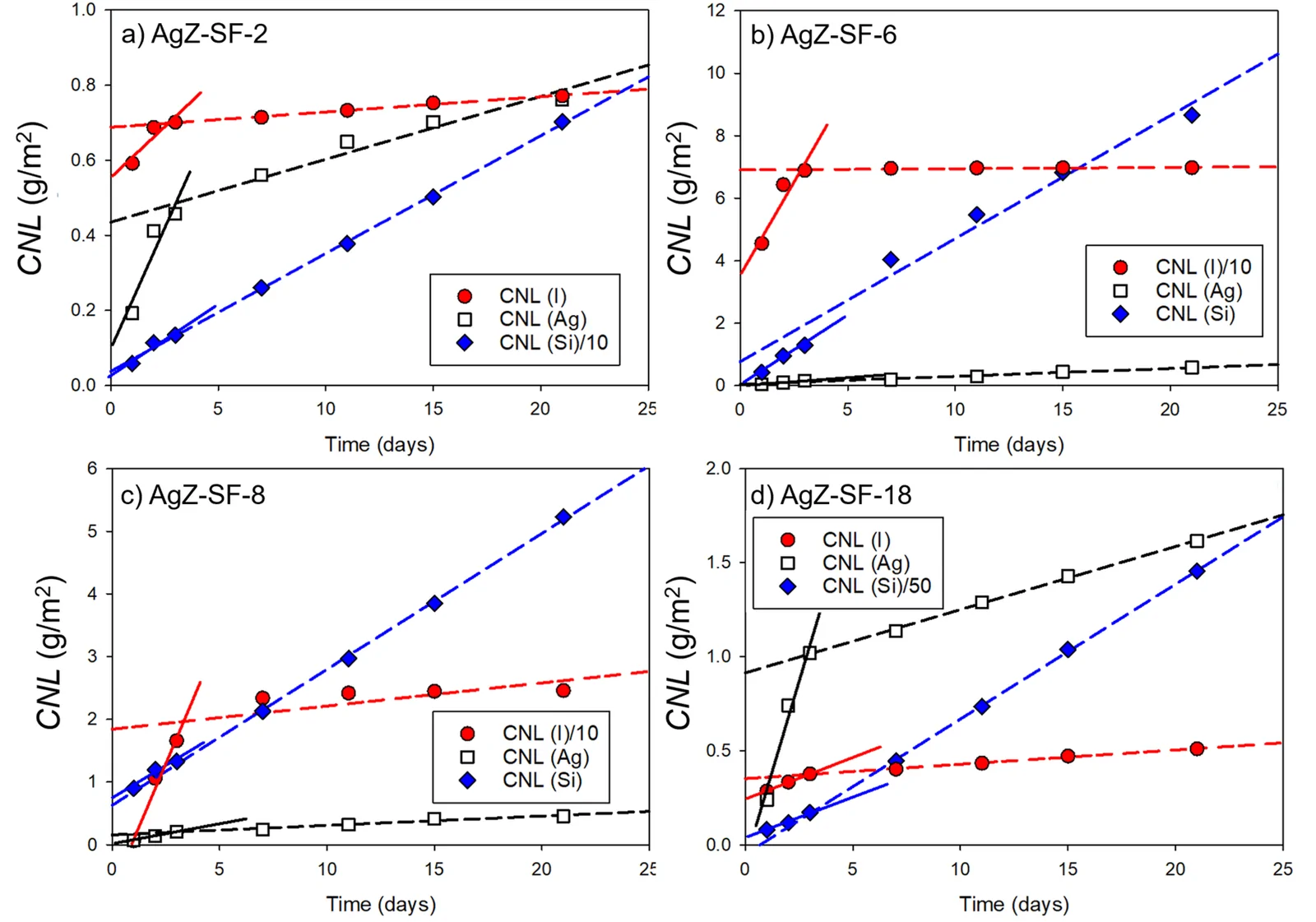
CNL-I, CNL-Ag, CNL-Si for (a) AgZ-SF-2, (b) AgZ-SF-6, (c) AgZ-SF-8, and (d) AgZ-SF-18 from the tests in Fig. 2. The CNL-I and CNL-Si values were adjusted by a factor of 10 or 50 for some plots (noted in plot legend) to provide an equal scale. Solid lines are drawn through the 1 day intervals and dashed lines through the longer (4 day and 6 day) intervals.

Further analysis of the corrosion processes during the leach tests were performed by comparing the CNL of each element (CNL-I, CNL-Ag, CNL-Si), as shown in Fig. 3. For each sample, regressions lines were determined separately for the short 1 day sample intervals (samples collected after 1, 2, and 3 days) and for the longer 4 day or 6 day sample intervals (samples collected after 7, 11, 15, and 21 days). This approach indicated that a higher corrosion rate occurred during the shorter intervals for the CNL-I and the CNL-Ag as the initially exposed host face dissolved and new host phase access was limited by the matrix. The CNL-Ag and CNL-I regression lines were not parallel to each other, likely due to the excess Ag that was not a part of the host phase in the waste form (*i.e.*, these sorbents did not have 100% use of Ag prior to HIP). However, the CNL-Si was consistent between the 1 day intervals and the longer (4 day and 6 day) intervals for all samples. A summary of the slopes of the regression lines is given in Table 4. These results indicate that the release of Si and corrosion of the matrix are under kinetic control by surface dissolution. However, the host phase undergoes a separate mechanism, likely based on solubility, and AgI corrosion was influenced by the changing interval duration.

**Table 4:** Linear regression slopes measured for the short (1 day) intervals and long (4 day and 6 day) intervals for the CNLs presented in Fig. 3. Values were calculated from raw data, not that adjusted by a factor of 10 or 50 in Fig. 3

Sample ID	1 day intervals (g m^−2^ d^−1^)			4 day and 6 day intervals (g m^−2^ d^−1^)		
	I	Ag	Si	I	Ag	Si
AgZ-SF-2	0.055	0.13	0.38	0.0048	0.018	0.30
AgZ-SF-6	12	0.05	0.61	0.014	0.031	0.35
AgZ-SF-8	0.80	0.07	2.2	0.014	0.022	2.2
AgZ-SF-18	0.046	0.39	2.3	0.0087	0.037	3.7

#### 3.2.2 Interval B sampling schedule

**Fig. 4:**
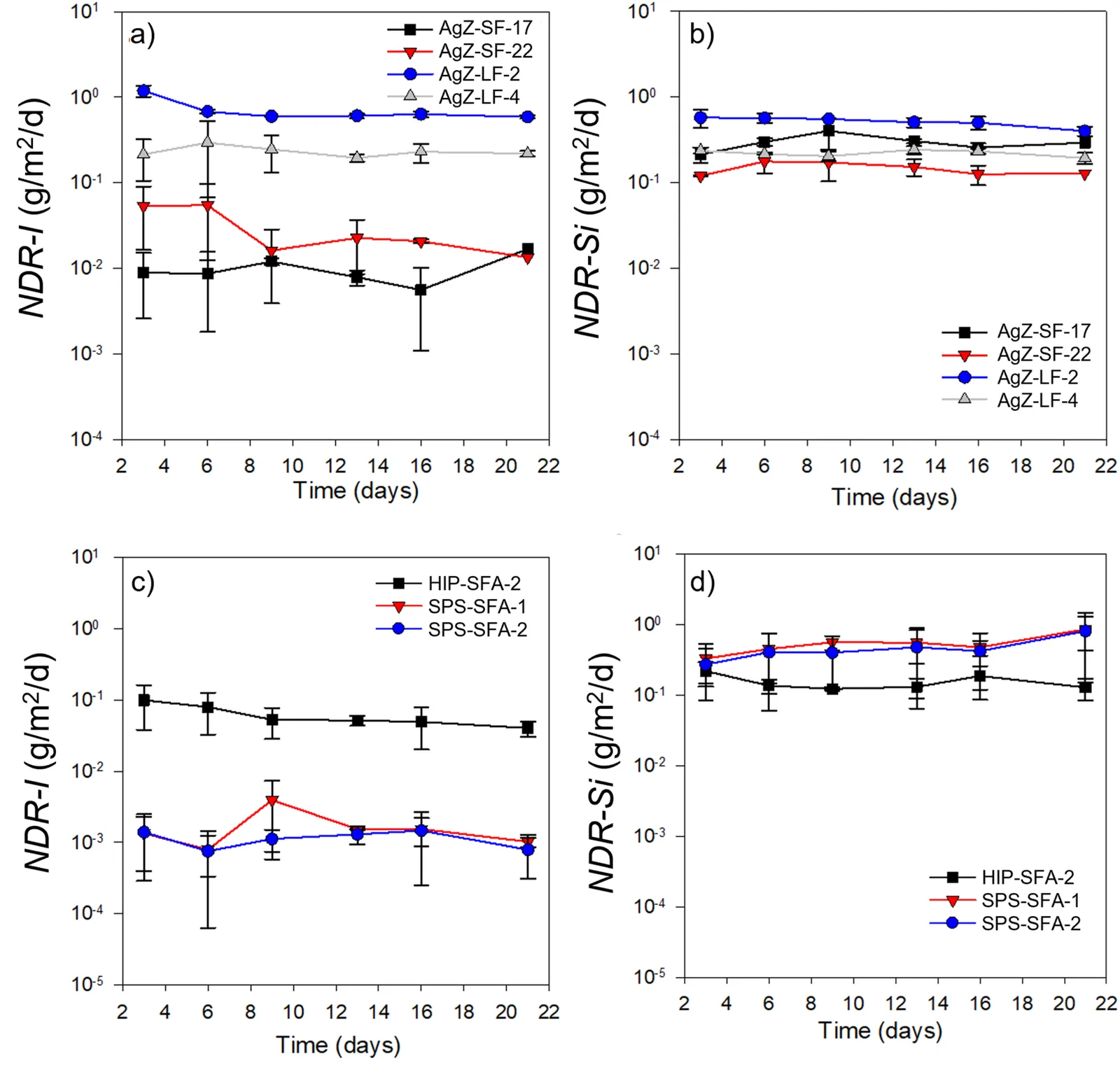
The (a), (c) NDR-I and (b), (d) NDR-Si measured for the (a), (b) SF and LF AgZ samples and for the (c), (d) HIP SFA and SPS SFA samples. Error bars show the standard deviation from the average of two replicates.

Semi-dynamic leach testing was performed using a regular interval set (Interval B) of 3, 6, 9, 12, 16, and 22 days on both AgZ and SFA samples (Fig. 4). A comparison between the results of the first sampling intervals is presented in the SI (Table S6) to show how the first samples could be applied as a single-test-interval screening test. The large-form AgZ-LF-4 sample (33% Ag use) had lower NDR-I (Fig. 4a) and NDR-Si (Fig. 4b) values compared to the AgZ-LF-2 sample (100% Ag use). Between the small-form samples, the AgZ-SF-17 sample (HIPed 12 h) had a higher NDR-Si but lower NDR-I at < 9 days than the AgZ-SF-22 sample (HIPed 3 h and dried). The measured NDR-I and NDR-Si for the HIPed AgZ samples were relatively consistent over length of the test, in contrast to the NDRs from the Interval A samples, suggesting dependence on interval length. The smaller AgZ samples (AgZ-SF) both had lower NDR-I than those of the larger samples (AgZ-LF). The AgZ-LF samples were larger in diameter, HIPed at a higher pressure (300 MPa) than the AgZ-SF samples (100 or 175 MPa), and the AgZ-LF-2 sample had higher iodine loading than the AgZ-SF samples. This observed trend suggests that the higher pressure used during HIPing may destabilize the host phase, similar to the influence of high temperatures observed in Fig. 2. The increase in corrosion rate with increased HIP temperature or increasing sample size warrants further investigation to determine the origin of the difference in NDR-I and to ensure IWF durability is not sacrificed when samples are scaled up.

As with the AgZ results, sample processing methods influenced trends in NDR-I (Fig. 4c) and NDR-Si (Fig. 4d) of the SFA samples. The SPS-SFA-1 and SFA-SPS-2 samples had a lower NDR-I (∼0.001 g m^−2^ d^−1^) compared to the HIP-SFA-2 sample (∼0.1 g m^−2^ d^−1^). The NDR-Si of the HIP and SPS samples were within the margin of error of each other. This observation highlights the improved iodine retention by SFAs that have been processed *via* SPS. The NDR-I and NDR-Si trends suggest that the encapsulating matrices in both the SPS and HIP samples are similar; however, the processing conditions in the HIP-SFA resulted in a more accessible host phase. The HIP method is a more rapid consolidation process than SPS, and the AgI is pushed to the edge of the SFA grain during HIPing.^38^ This, combined with the lower porosity (higher density) of the HIP waste form (determined in previous work^39^), may result in leachant having greater access to the AgI host phase and thus weaker corrosion resistance.

**Fig. 5:**
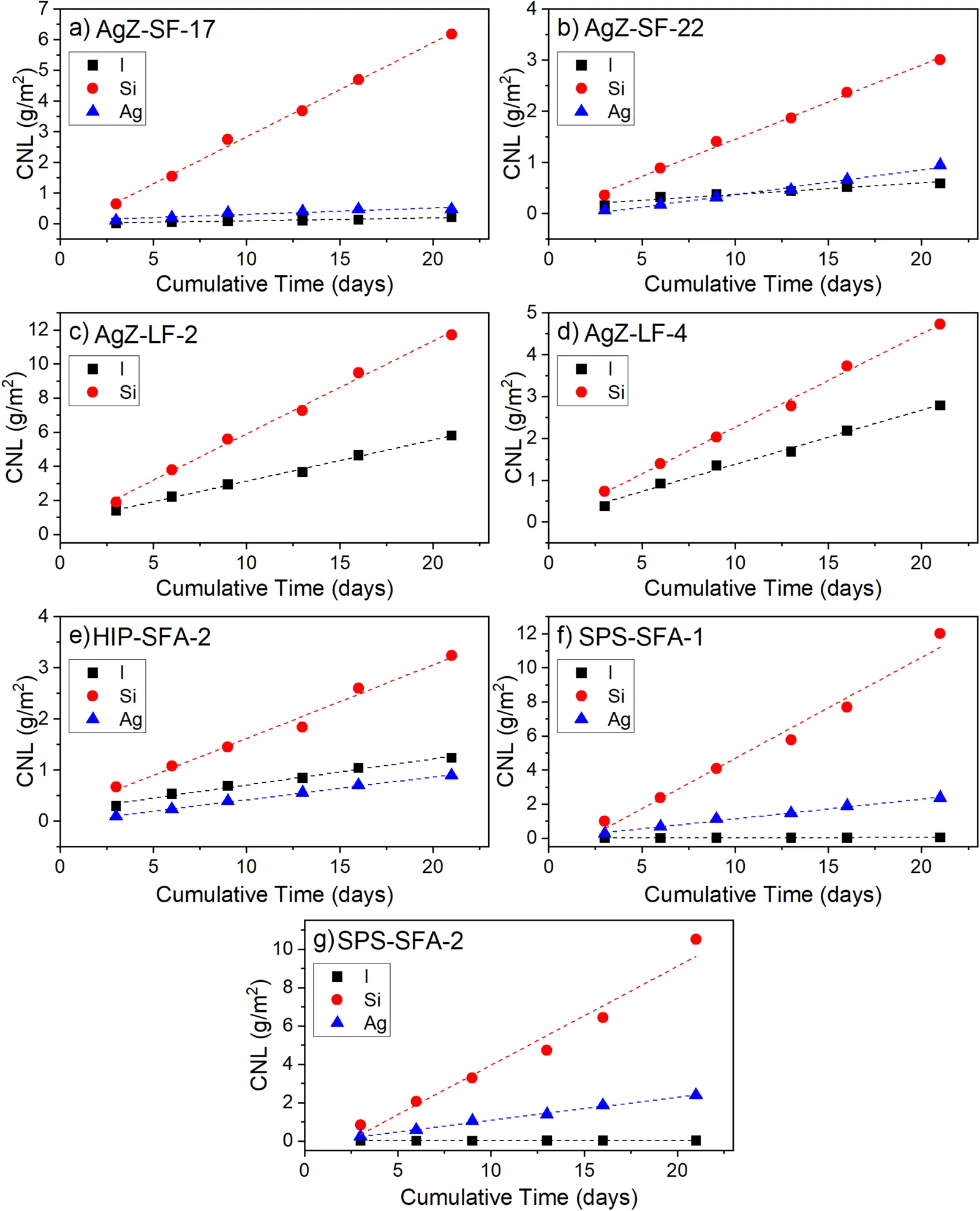
Comparison of CNL over time using the average values from Fig. 4 for the (a) AgZ-SF-17, (b) AgZ-SF-22, (c) AgZ-LF-2, (d) AgZ-LF-4, (e) HIP-SFA-2, (f) SPS-SFA-1, and (g) SPS-SFA-2 samples.

A comparison of the CNL for the Interval B semi-dynamic leach tests is shown in Fig. 5 for the AgZ and SFA samples. The slopes from the regression lines are listed in Table 5. The slopes for the AgZ-LF samples were much larger for iodine than they were for the AgZ-SF samples, indicating that changes in AgZ sample size may have influenced corrosion of the host phase. The slopes are also constant across the entire test period using Interval B, unlike the variation seen in Interval A with different slopes for the short (1 day) intervals and long (4 day and 6 day) intervals. The Ag values were below the ICP-OES detection limits (26.4 or 53.7 µg L^−1^, depending on the dilution of the sample) for the AgZ-LF samples and therefore were not included. The CNL slopes of iodine corrosion tests with HIPed SFAs were similar to those of tests with AgZ-SFs, and those of SPS SFAs were a little lower.

**Table 5:** Slopes of regression fits for the data in Fig. 5. Slopes labeled “ND” are included to account for species below the ICP-OES detection limit (*i.e.*, “not detected”) in the leachates of that test. The detection limit for Ag was 26.4 or 53.7 µg L^−1^, depending on dilution of the sample

Waste form type	Sample ID	Interval regression (g m^−2^ d^−1^)		
		I	Si	Ag
HIP AgZ	AgZ-SF-17	0.010	0.31	0.021
	AgZ-SF-22	0.022	0.15	0.049
	AgZ-LF-2	0.24	0.54	ND
	AgZ-LF-4	0.13	0.22	ND
SFA	SFA-HIP-2	0.051	0.14	0.044
	SFA-SPS-1	0.0018	0.59	0.12
	SFA-SPS-2	0.0011	0.52	0.12

#### 3.2.3 Temperature effects

**Fig. 6:**
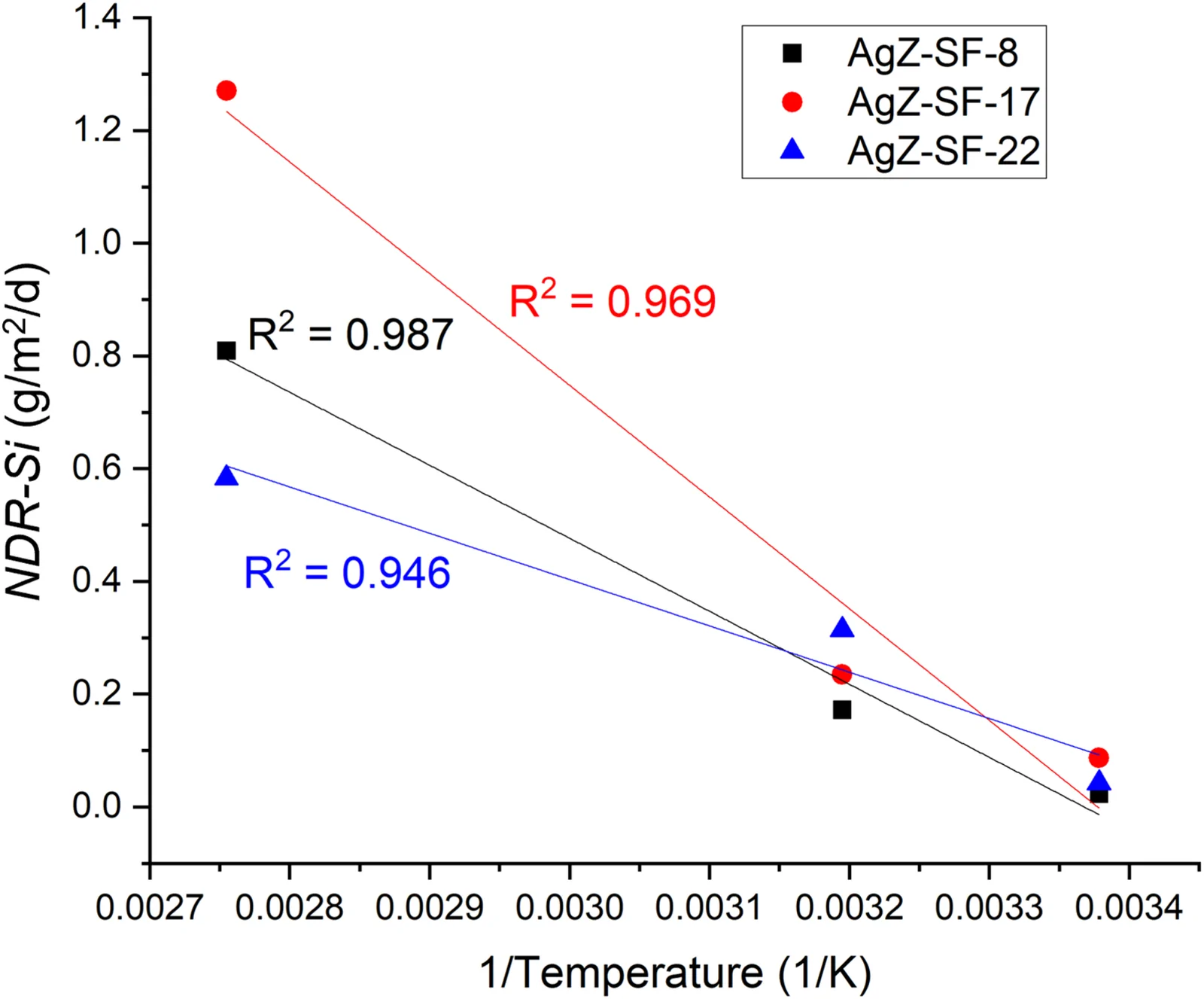
Plot showing the NDR-Si from a single 1 day interval against the inverse temperature at which the test was performed. The *R*^2^ values correspond to the specific samples analyzed.

Most IWF corrosion testing is carried out at 90 °C to ensure data could be collected in reasonable test times. An evaluation of alternate temperatures was performed using AgZ-SF samples. A temperature dependence of the dissolution rate of the matrix or host phase would indicate an activation energy term being required in a CCRM to allow extrapolations of the dissolution rates to the evolving repository temperatures. Table 6 provides the resulting NDR-I and NDR-Si for samples after a 1 day interval at different temperatures. The tests at room temperature (23 °C) had no measurable iodine; however, the silicon concentrations were measurable. An Arrhenius plot of the NDR-Si *vs.* reciprocal test temperature (in K) was generated (Fig. 6) and suggests an Arrhenius relationship exists for the matrix dissolution rates. Longer test intervals are required at lower temperatures to attain measurable values. A balance between test temperature and a common ion effect on the waste form dissolution requires further study. Because dissolution rate decreased with decreasing temperature, an activation energy term should be added to any CCRM.

**Table 6:** Comparison of the NDR for samples tested at multiple temperatures in a 1 day interval for iodine (NDR-I) and silicon (NDR-Si)^a^

Sample ID	Temperature (°C)	NDR-I (g m^−2^ d^−1^)	NDR-Si (g m^−2^ d^−1^)
AgZ-SF-8	23	ND	2.32 × 10^−2^
	40	3.14 × 10^−1^	1.72 × 10^−1^
	90	5.36 × 10^−1^	8.09 × 10^−1^
AgZ-SF-17	23	ND	8.74 × 10^−2^
	40	5.39 × 10^−2^	2.35 × 10^−1^
	90	1.45 × 10^1^	1.27 × 10^0^
AgZ-SF-22	23	ND	4.21 × 10^−2^
	40	1.03 × 10^−1^	3.14 × 10^−1^
	90	1.23 × 10^0^	5.84 × 10^−1^

a ND = non-detect (below instrument detection limits).

#### 3.2.4 Leachant replacement

**Fig. 7:**
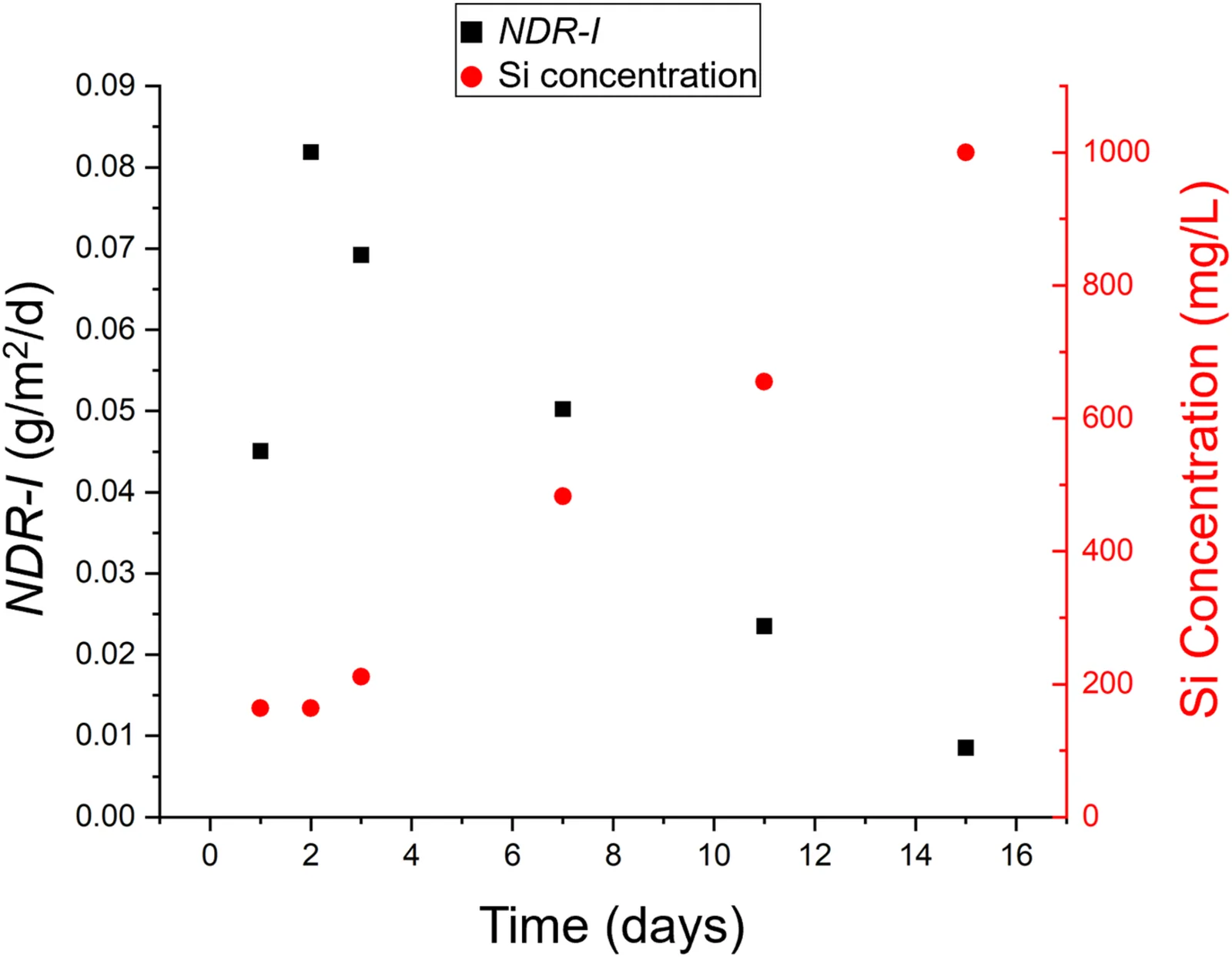
The normalized dissolution rate (NDR-I) measured for the AgZ-LF-3 sample in a static leach test. The corresponding Si concentration within the leachate is displayed on the right.

During the dissolution of materials with affinity-based kinetics, a common ion effect can occur in which the hydrolysis reaction is slowed due to the accumulation of the resulting dissolved species in solution.^32^ For an IWF, this could look like the slowing of the dissolution of the matrix as NDR-Si, in turn slowing the exposure of the host phase and slowing the release of iodine. If this process occurs for a specific type of IWF, it should be captured in a CCRM. A preliminary investigation of this effect on a HIP AgZ sample (AgZ-LF-3) was performed in DDI at 90 °C using Interval A. Instead of a full leachant replacement at each interval, the leachant was static, and a small volume (250 µL) of leachate was collected at each interval to minimize changing the *S*/*V* ratio. The AgZ is primarily made of Si, and accumulation of Si in solution could slow the dissolution of the matrix. The resulting NDR-I is shown in Fig. 7 along with the measured Si concentration in the leachate. The NDR-I was determined by the difference in iodine concentration between each interval. Through the test intervals, the Si concentration continually increased while the NDR-I decreased. The NDR-I after 15 days in the static test was 0.008 g m^−2^ d^−1^. The NDR-I of AgZ-LF-4 after 16 days in the semi-dynamic leach test was significantly higher at 0.13 ± 0.005 g m^−2^ d^−1^ (Fig. 4). AgZ-LF-4 was a replicate of AgZ-LF-3 assuming any inherent microstructural differences between two samples processed from the same material under identical conditions had minimal influence on the dissolution rates. This observation suggests that a common ion effect may have occurred and slowed the dissolution of the host phase. A similar observation of the common ion effect on IWFs was made previously on sodalite-based IWFs.^40^ The extent of this common ion effect on various IWF types is currently under further investigation to determine if an affinity term to represent the common ion effect is required in an IWF CCRM. Testing is required where the concentration of components which are released from the IWF (*e.g.*, Si, Al) are systematically varied and the resulting NDR-Si calculated.

#### 3.2.5 Leachant pH

**Fig. 8:**
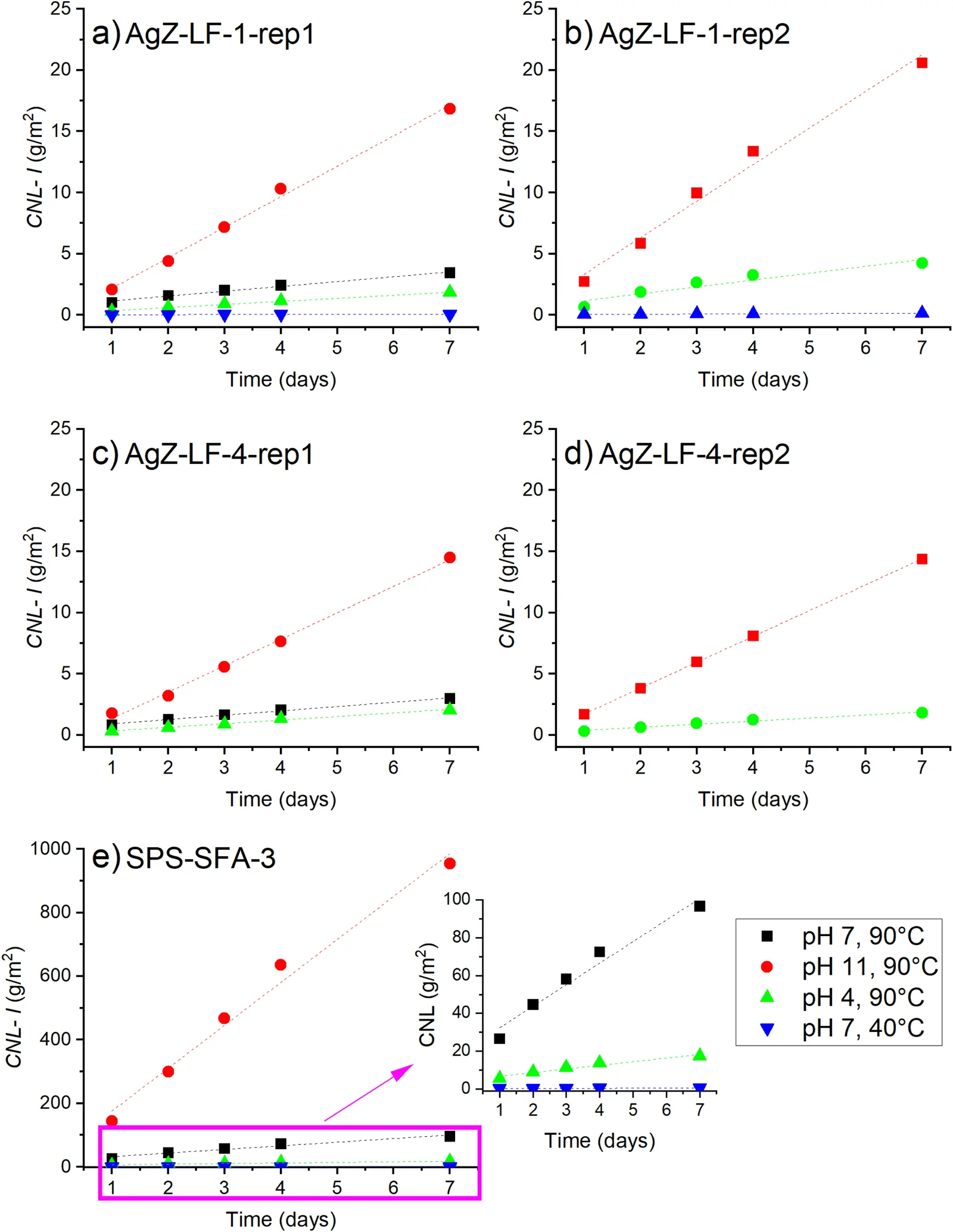
CNL of iodine for AgZ and SPS SFA samples during semi-dynamic leach tests at various pH and temperatures. (a) and (b) Are replicates (rep) of AgZ-LF-1 (100% Ag use) and (c) and (d) are replicates of AgZ-LF-4 (33% Ag use). (e) Shows the CNL-I of SPS-SFA-3.

**Fig. 9:**
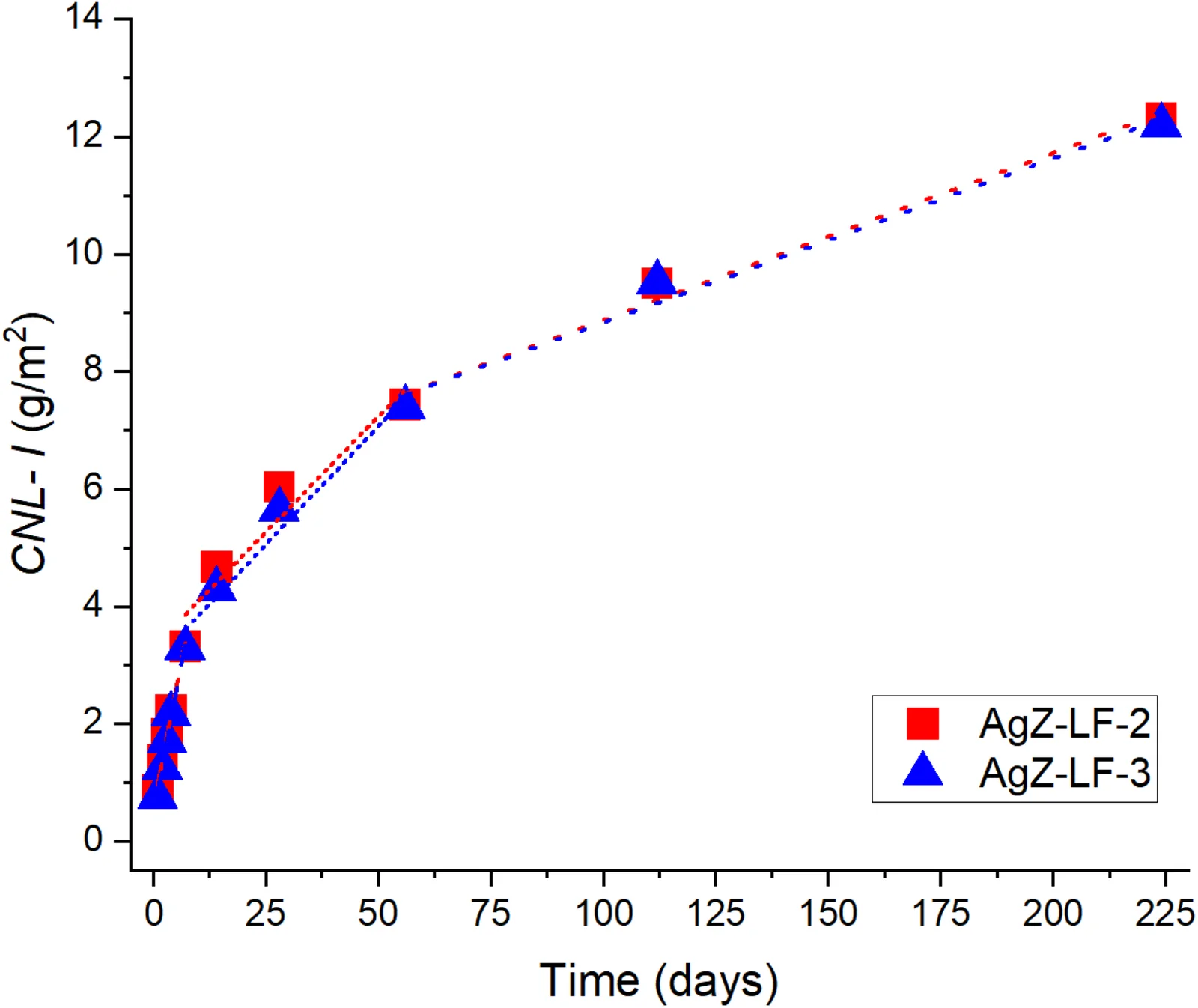
CNL of iodine from semi-dynamic leach tests performed at 90 °C and pH 7 on AgZ-LF-2 (100% Ag use) and AgZ-LF-3 (33% Ag use) following Interval D.

To understand the influence of pH on IWF corrosion rates, semi-dynamic leach tests were performed at pH 4 (90 °C), pH 7 (40 °C and 90 °C), and pH 11 (90 °C) following sampling schedules Interval C (Fig. 8) and Interval D (Fig. 9). The HIP AgZ samples had relatively consistent dissolution rates measured across sample replicates at each condition, confirming repeatability of the test method (Table 7). The dissolution rates were highest at pH 11 and similar at pH 4 and pH 7. The origin of the consistency between pH 4 and pH 7 is unknown but the silica-based network has been shown to have a V-type behavior around neutral pH.^41^ IWF corrosion is likely pH-dependent and this should be represented in the CCRM. Further work should be performed to help determine the role of pH on IWF corrosion and define a pH term for the numerical waste form degradation model.

**Table 7:** NDR of iodine averaged over testing period days 1–7 (Interval C) or days 1–7, 7–56, and 56–224 (Interval D). Replicate (abbreviated “rep”) samples are labeled as replicate 1 or 2^a^

NDR-I for tests performed with interval C (g m^−2^ d^−1^)					NDR-I for tests performed with interval D (g m^−2^ d^−1^)			
Temperature	90 °C	90 °C	90 °C	40 °C	Temperature	90 °C	90 °C	90 °C
pH	7	11	4	7	pH	7	7	7
AgZ-LF 1-rep1	0.39 ± 0.02	2.49 ± 0.10	0.25 ± 0.01	0.007 ± 0.001	Test period	1–7 days	7–56 days	56–224 days
AgZ-LF 1-rep2	N/A	2.99 ± 0.20	0.56 ± 0.10	0.016 ± 0.001	AgZ-LF-2	0.42 ± 0.02	0.08 ± 0.01	0.03 ± 0.003
AgZ-LF 4-rep1	0.35 ± 0.02	2.16 ± 0.08	0.29 ± 0.02	ND				
AgZ-LF-4-rep2	N/A	2.11 ± 0.01	0.25 ± 0.02	ND	AgZ-LF-3	0.40 ± 0.02	0.08 ± 0.02	0.03 ± 0.003
SPS-SFA-3	11.4 ± 1.22	135 ± 9.32	1.92 ± 0.26	0.058 ± 0.008				

a N/A: data not available, ND: non-detect, data below detection limit.

The SPS SFA sample (Table 7) had higher dissolution rates than the AgZ samples, most markedly at pH 11. Decreasing the test temperature from 90 °C to 40 °C at pH 7 led to a decrease in dissolution rate by at least an order of magnitude. At the lower temperature, challenges were encountered with many leachate concentrations being below the instrument detection limit and, as such, longer intervals may be needed in future efforts to evaluate the influence of test temperature. The same challenge was encountered with the other elements of interest (*i.e.*, Si, Al, Ag), making comparisons between the host phase and matrix dissolution limited.

Long-term test data is required to evaluate the ability of the numerical waste form degradation model to replicate corrosion data. A pair of long-term tests at pH 7 and 90 °C were initiated on AgZ-LF-2 and AgZ-LF-3 samples using Interval D. The resulting NDR and CNL of iodine are presented in Table 7 and Fig. 9. There were three regimes in the long-term tests. In the first, the NDR-I of the first 7 days of testing was similar to that of tests performed over 7 days (Interval C). In the second regime, the NDR-I was ∼8 times lower between days 7 and 56. By the third regime (days 56–224), the dissolution rate further decreased. This decrease in the NDR-I was likely due to the common-ion effect slowing dissolution with time in the longer intervals as dissolved components from the IWF built up in solution and the initial surface iodine depleted. Following corrosion testing, sample surfaces were imaged for changes in the microstructure (SI Fig. S15–S17). Little change was observed, and further characterization is needed to assess the extent of selective etching across pH regimes at extended test times.

#### 3.2.6 Other effects

Changing the remaining alternate test variables (*S*/*V* ratio, masking, and surface finish) had minor effects on the observed NDR-I and NDR-Si of the various waste forms. The NDR-I tended to decrease somewhat with increasing *S*/*V* ratios but not at equal changes in magnitude. For the SPS-SFA-2 test, tripling the *S*/*V* ratio to 0.3 cm^−1^ resulted in an NDR-I that decreased to 70% of the NDR-I of the original test with a 0.1 cm^−1^*S*/*V* ratio. When decreasing the *S*/*V* 10-fold, the NDR-I was about 3 times larger for AgZ-LF-2 (100% Ag use) and 6 times larger for AgZ-LF-4 (100% Ag use). Quantitative data are presented in the SI (Table S7), but the results suggest that increasing the *S*/*V* ratio for a leach test (and thus likely needing a larger vessel) is unnecessary given the minor changes associated with modifying the *S*/*V* ratio.

The NDR-I of both the HIP and SPS SFA samples were nearly identical with and without a mask (Fig. S18). Meanwhile, the NDR-Si of the HIP SFA was an order of magnitude greater than that of the unmasked sample. This may have been due to contamination from an uncured RTV silicone mask or instability of the mask at test temperatures.

For the replicate AgZ-LF-1 and AgZ-LF-2 samples, the NDR-I and NDR-Si were similar for all surface finish treatments except the 1200 grit finish having a slightly higher NDR-I (Fig. S19). For the AgZ-LF-3 and AgZ-LF-4 replicate samples, the finer polished samples had slightly lower NDR-I values and similar NDR-Si values. The results of the surface finish testing suggest that the 600-grit polish applied for most tests in the present study can still be used to minimize sample loss during repolishing for reuse of samples, minimize dissolution of AgI, and minimize the number of polishing steps required to prepare the samples.

### 3.3 Post-corrosion sample characteristics

SEM images were collected both before and after semi-dynamic leach tests to observe any evolution of the sample microstructure. The same area on the sample surface was imaged when possible. Samples AgZ-SF-2, AgZ-SF-17, and AgZ-SF-22 showed little to no change in their microstructures following testing, and their corresponding images are presented in the SI (Fig. S20–S22). Samples AgZ-SF-6 and AgZ-SF-8 (both HIPed at 900 °C) had a newly exposed host phase region of AgI while AgZ-SF-18 (HIPed at lowest temperature of 525 °C) displayed extensive matrix phase corrosion (Fig. S23). Although not performed in the present study, previous research using optical profilometry of AgZ and SFA samples showed that corrosion damage during leach testing is minor around pH 7 and may increase at higher pH values.^22^

The AgZ-LF samples were HIPed at 900 °C and did not display large areas of host phase exposure following corrosion. The green regions (outer ring of AgZ-LF-1 and AgZ-LF-2, distributed throughout AgZ-LF-3 and AgZ-LF-4) were corroded to expose more of the AgI host phase (Fig. S24).

The SPS SFA samples had much lower NDR-I values than their HIP SFA counterparts. However, minimal changes to the matrix phases were observed on the SPS SFA surface (Fig. S25). This observation aligns with the behavior shown in Fig. 4c and d where the HIP SFA has a higher NDR-I from the dissolving AgI host phase and a similar NDR-Si to the SPS SFA due to the matrix durability.

## 4 Summary and conclusions

The development of a CCRM for IWFs requires a consistent test method to assess IWF corrosion and an understanding of the corrosion mechanisms of the IWFs of interest. The work within this study advanced both areas by presenting information on the differences in corrosion of two IWF types based on their processing history using semi-dynamic leach tests. This testing involved semi-dynamic leach testing of monolithic IWF samples (with changes to sampling interval, temperature, leachant replacement, leachant pH, surface-area-to-volume ratio, masking, and surface finish) and was applied to HIP AgZ, HIP SFA, and SPS SFA samples.

The HIP conditions of the AgZ samples directly influenced the durability of the waste form. At low HIP temperatures of 525 °C, the matrix of the AgZ sample was more susceptible to corrosion attack, causing more host phase to be exposed with time. Samples HIPed at 900 °C had high initial NDR-I values and lower matrix dissolution as the high temperature may have destabilized the AgI host phase while simultaneously stabilizing the matrix phase. Testing of large form AgZ samples showed higher NDR-I values compared with the smaller AgZ samples prepared under the same conditions. The temperature profile within the canister during HIPing of the large form AgZ samples differed from that in the small form AgZ samples, possibly leading to variations in microstructures and overall IWF corrosion behavior.

A comparison was also made between SFA samples that were densified using either HIP or SPS. The HIP SFA had a higher NDR-I compared to that of the SPS SFA. Images of the microstructure following corrosion showed a depletion of the AgI host phase from the HIP SFA sample and some increased exposure of the AgI host phase in the SPS SFA sample. Despite the difference in host phase behavior and iodine release, the two sample types had similar NDR-Si, suggesting equally durable matrix phases. The processing method for SFA has a direct effect on AgI host phase stability. When SFAs were prepared by HIP, the porosity increased and the AgI was pushed to the boundary of the SFA grain, which made the AgI host phase more accessible and reactive.

Altering the *S*/*V* ratio, masking, and surface finish of the IWF had minor impacts on the NDR-I and NDR-Si of the various IWFs and could be varied as required to meet the needs of unique IWF types. Temperature, leachant replacement, and leachant pH had a larger impact on iodine dissolution and should be represented in a CCRM. The NDR-I decreased with decreasing temperature. When the leachant was not replaced (which could simulate the accumulation of dissolved IWF components in a waste repository), a common ion effect occurred and slowed dissolution of the host phase. IWF corrosion was higher at pH 11 than at pH 7 or pH 4, and long-term corrosion testing performed over 224 days showed that the iodine dissolution rate decreased over time. Whether this slowing trend continues is not known as data remains limited on long-term corrosion behavior of IWF. From the experimental matrix assessed in the present study, a suggested standardized leach-test protocol would follow Interval B (less sensitive to fluctuations in early stages of leach testing, might give better information about long-term contaminant behavior), 90 °C (to collect data in a reasonable time frame), pH 7 (because DDI pH may fluctuate as it equilibrates with the atmosphere or shift with the addition of the waste form), a *S*/*V* ratio of 0.1 cm^−1^, masked, and polished with 600 grit.

While the progress in measuring corrosion rates and understanding IWF behavior in this work is substantial, some technical gaps remain. Applying similar tests to a wide range of IWF types (including those that are not silica-based or aluminosilicate-based), would allow head-to-head comparisons of chemical durability between IWF types as test variables are consistent, similar to the work of Reiser *et al.*^18^ The current test suite uses DDI and pH buffers as a leachant, but the test suite should also be evaluated using simulated repository waters (*e.g.*, simulated to have repository-relevant salinity, bicarbonate and/or SiO_2_ concentrations) to assess any differences induced as a result similar to the approach suggested in the MCC-1 method.^42^ Infiltrating water in repositories will be more chemically complex and could alter corrosion behavior (*e.g.*, the common ion effect was observed on the AgZ samples in the present study). Studies on other IWFs have reported higher corrosion rates with increasing ionic strength^43^ and ion exchanges between IWFs and other anions in the groundwater affecting iodine behavior.^44^ The extent of the common ion effect controlling IWF corrosion and which components released from the waste form have the largest impact should also be pursued. As a CCRM for IWF is developed, it is likely that similar testing could be used for parameterizing the CCRM.

## Author contributions

Amanda R. Lawter: formal analysis, investigation, methodology, project administration, writing – original draft, writing – reviewing & editing. Gemma G. Clark: formal analysis, writing – original draft, writing – reviewing & editing. Nancy M. Avalos: formal analysis, investigation. Jeff Bonnett: investigation. Nathan Canfield: investigation. Seungrag Choi: formal analysis, investigation, writing – original draft, writing – reviewing & editing. Mark E. Bowden: formal analysis. Josef Matyas: resources. R. Matthew Asmussen: conceptualization, formal analysis, funding acquisition, investigation, methodology, project administration, supervision, writing – original draft, writing – reviewing & editing.

## Conflicts of interest

There are no conflicts to declare.

## Supplementary Material

RA-016-D6RA01079B-s001

## Data Availability

Data supporting this article have been included as part of the supplementary information (SI). Additional data are available upon request from the authors. Supplementary information: a summary of common leaching interval test methods, composition details and XRD characterization of the IWF samples, SEM/EDS maps and photographs of samples, and discussion of other test variables. See DOI: https://doi.org/10.1039/d6ra01079b.
