# Supplementary material for: Semi-dynamic leach testing of densified silicon-based iodine waste forms
Source: RSC Adv. 2026 Apr 17;16(22):19974–90. doi: 10.1039/d6ra01079b (PMC13089361; doi:10.1039/d6ra01079b)
Supplement: RA-016-D6RA01079B-s001 [file RA-016-D6RA01079B-s001.pdf]

Supplementary Information (SI) for RSC Advances.
This journal is © The Royal Society of Chemistry 2026

Supplementary information for

Semi-Dynamic Leach Testing of Densified Silicon-Based Iodine Waste Forms

Amanda R. Lawter,[#] Gemma G. Clark,[#] Nancy Escobedo, Jeff Bonnett, Nathan Canfield, Seungrag Choi, Mark E. Bowden, Josef Matyas, R. Matthew Asmussen[*]

Pacific Northwest National Laboratory (PNNL), 902 Battelle Boulevard, Richland, WA 99352

# authors contributed equally

*corresponding author

Address: Pacific Northwest National Laboratory (PNNL), P.O. Box 999, MSIN P7-54, Richland, WA 99352

E-mail: matthew.asmussen@pnnl.gov

Phone: (509) 371-7223

Fax: (509) 371-7249

S.1 Common leaching interval test methods

A comparison of the leaching intervals of common test methods is shown in Table S1. ASTM Method C1220-17 includes four interval types. Each interval matrix had a different purpose with the intention that, when used together, these matrices could be used to compare different materials and their behaviors. The Materials Characterization Center-1 (MCC-1) method, which has been replaced by the ASTM Method C1220, suggests suspending a monolithic sample in a brine, water, or silicate solution and collecting samples at varying intervals over a 28-day period. Each of these interval sets could induce a unique response in IWF behavior.

Table S1. Summary of methods for semi-dynamic leach testing in hours (h) and days (d).

**EXAMPLE PUBLISHED LEACHING TEST METHODS**		
**Test Method**	**Description**	**Sampling schedule, cumulative time**
ASTM Method C1308-08 (2017)	Accelerated test for measuring contaminant releases from solidified waste	2h, 5h, 17h, 1d, 2d, 3d, 4d, 5d, 6d, 7d
ASTM Method C1308-21 (2021)	Same as above, updated version	1d, 2d, 3d, 4d, 5d, 6d, 7d, 8d, 9d, 10d, 11d
ANS/ANSI 16.1 (2003)	Measurement of the leachability of solidified low-level radioactive wastes	2h, 7h, 1d, 2d, 3d, 4d, 5d (abbreviated test)<br>2h, 7h, 1d, 2d, 3d, 4d, 5d, 19d, 47d, 90d (extended test)
ANS/ANSI 16.1 (2019)	Same as above, updated version	1d, 2d, 3d, 4d (additional 1-day intervals optional)
ASTM Method C1220-17	Static leaching of monolithic waste forms – matrix of four separate tests with four different sampling intervals	7d (screen test)<br>28d (inter-laboratory test)<br>3d, 7d, 14d, 28d, 56d, 91d, 192d, 364d (solution saturation test)<br>16h, 24h, 40h, 2d, 3d, 4d, 5d, 6d, 7d, 10d, 14d (material dissolution test)
EPA Method 1315	Semi-dynamic leach test	2h, 1d, 2d, 7d, 14d, 28d, 42d, 49d, 63d
MCC-1	Leach test for nuclear waste forms	28d (brine test)<br>3d, 7d, 14d, 28d (water test)<br>28d (silicate test)

S.2 IWF sample composition

Table S2. Composition of IWF samples determined by EDS in wt%. The error presented for each value is the standard deviation (±1σ) from multiple sample analyses. Samples without standard deviation values did not have multiple analyses performed. For AgZ-LF samples, the error includes differences between areas of different appearance from each coupon. "ND" indicates non-detect.

Element (wt %)	AgZ-SF-2	AgZ-SF-6	AgZ-SF-8	AgZ-SF-17	AgZ-SF-18	AgZ-SF-22	AgZ-LF-1	AgZ-LF-2	AgZ-LF-3	AgZ-LF-4	HIP-SFA-1	HIP-SFA-2	SPS-SFA-1	SPS-SFA-2	SPS-SFA-3
**O**	47 ± 3.1	46 ± 0.6	45 ± 1.2	47 ± 1.2	49 ± 1.9	47 ± 2.8	45 ± 1.7	47 ± 0.7	46 ± 2.6	48 ± 2.8	32	36	39	39	33
**Na**	0.3 ± 0.0	0.4 ± 0.0	0.3 ± 0.1	0.5 ± 0.0	0.3 ± 0.0	0.4 ± 0.0	0.1 ± 0.0	0.1 ± 0.0	0.1 ± 0.0	0.2 ± 0.1	ND	ND	ND	ND	ND
**Mg**	0.7 ± 0.0	0.7 ± 0.1	0.7 ± 0.0	0.8 ± 0.1	0.4 ± 0.2	0.6 ± 0.0	0.5 ± 0.0	0.5 ± 0.0	0.5 ± 0.1	0.5 ± 0.1	ND	ND	ND	ND	ND
**Al**	6.0 ± 0.3	5.7 ± 0.3	5.8 ± 0.1	5.9 ± 0.1	4.2 ± 1.3	5.7 ± 0.2	5.6 ± 0.3	5.8 ± 0.1	5.4 ± 0.3	5.9 ± 0.3	0.05	0.0	0.0	0.0	ND
**Si**	31 ± 0.6	30 ± 1.2	31 ± 1.1	32 ± 0.1	33 ± 2.5	30 ± 1.7	33 ± 1.4	33 ± 0.6	32 ± 1.4	33 ± 1.0	26	28	34	34	30
**S**	ND	ND	ND	ND	ND	ND	ND	ND	ND	ND	ND	0.3	1.2	1.0	ND
**K**	1.1 ± 0.0	1.1 ± 0.1	0.9 ± 0.0	1.0 ± 0.1	0.6 ± 0.1	1.0 ± 0.0	0.4 ± 0.0	0.4 ± 0.0	0.5 ± 0.2	0.6 ± 0.2	ND	ND	ND	ND	ND
**Ca**	1.3 ± 0.2	1.2 ± 0.1	1.4 ± 0.1	1.2 ± 0.1	1.0 ± 0.3	1.2 ± 0.1	0.6 ± 0.1	0.6 ± 0.1	0.7 ± 0.2	0.8 ± 0.2	ND	ND	ND	ND	ND
**Fe**	0.7 ± 0.0	0.6 ± 0.0	0.7 ± 0.0	0.7 ± 0.0	0.5 ± 0.1	0.7 ± 0.1	2.5 ± 1.2	1.2 ± 0.2	1.4 ± 0.2	0.9 ± 0.1	ND	ND	ND	ND	ND
**Ag**	7.9 ± 0.5	7.7 ± 0.0	7.8 ± 1.0	6.0 ± 0.7	6.1 ± 0.4	9.5 ± 1.0	5.9 ± 1.3	5.4 ± 0.7	7.5 ± 1.9	6.6 ± 1.3	21	21	16	16	7
**I**	6.3 ± 0.1	5.0 ± 0.3	6.1 ± 1.5	5.0 ± 0.4	4.6 ± 2.0	3.1 ± 0.4	5.9 ± 1.3	5.3 ± 0.7	6.3 ± 2.7	3.6 ± 3.2	13	15	9.8	11	30

S.3 Sample composition using nominal starting iodine concentration

The following table presents the composition of the AgZ-LF and SFA aerogel samples measured using EDS but adjusted for the starting concentration of iodine prior to processing and analysis. These compositions were used to determine the normalized dissolution rate (*NDR*) values in Section S.4.

Table S3. Composition of the samples used in the study determined by EDS and adjusted for the nominal starting amount of iodine assuming no loss to volatilization during processing or EDS. These compositions were used to determine the *NDR* in the plots presented in Figure S1 and Figure S2.

**Element (wt %)**	**AgZ-LF-1**	**AgZ-LF-2**	**AgZ-LF-3**	**AgZ-LF-4**	**SPS-SFA-1**	**SPS-SFA-2**	**HIP-SFA-2**
**O**	43	43	46	47	30	30	27
**Na**	0.1	0.1	0.2	0.2	0.0	0.0	0.0
**Mg**	0.4	0.4	0.5	0.5	0.0	0.0	0.0
**Al**	5.3	5.4	5.7	5.8	0.0	0.0	0.0
**Si**	30	30	32	32	26	26	22
**S**	-	-	-	-	0.9	0.8	-
**K**	0.4	0.4	0.5	0.6	-	-	-
**Ca**	0.6	0.6	0.8	0.7	-	-	-
**Fe**	1.0	1.2	1.0	0.8	-	-	-
**Ag**	5.2	4.8	6.8	6.1	13	12	17
**I**	14	14	6.4	6.4	30	30	30

S.4 *NDR* calculated using nominal iodine starting concentrations

This section provides the *NDR-I* and *NDR-Si* adjusted for the compositions in Table S3 using the nominal starting concentrations of iodine.

Table S4. Normalized dissolution rate, with respect to iodine and silicon, for the various samples evaluated in the single sampling 3-day screening test. Measurements with an associated error were performed in duplicate (and the AgZ-SF-8 sample without an error presented was not).

IWF Type	Sample ID	NDR-I ($g/m^2/d$)	NDR-Si ($g/m^2/d$)
Ag-mordenite (small)	AgZ-SF-8	1.8×10^{-2}	6.6×10^{-2}
	AgZ-SF 17	$8.9 \pm 6.3\times10^{-3}$	$2.1 \pm 0.4 \times10^{-1}$
	AgZ-SF-22	$5.4 \pm 3.7\times10^{-2}$	$1.2 \pm 0.001 \times10^{-1}$
Ag-mordenite (large)	AgZ-LF-2	$4.7 \pm 0.9 \times10^{-1}$	$6.3 \pm 1.5\times10^{-1}$
	AgZ-LF-4	$1.3 \pm 0.9 \times10^{-1}$	$1.3 \pm 0.8 \times10^{-1}$
Ag-functionalized silica aerogels	HIP-SFA-2	$9.9 \pm 6.1\times10^{-2}$	$2.2 \pm 0.7 \times10^{-1}$
	SPS-SFA-1	$1.3 \pm 0.9 \times10^{-3}$	$3.3 \pm 2.0 \times10^{-1}$
	SPS-SFA-2	$1.4 \pm 1.1\times10^{-3}$	$2.8 \pm 1.9 \times10^{-1}$

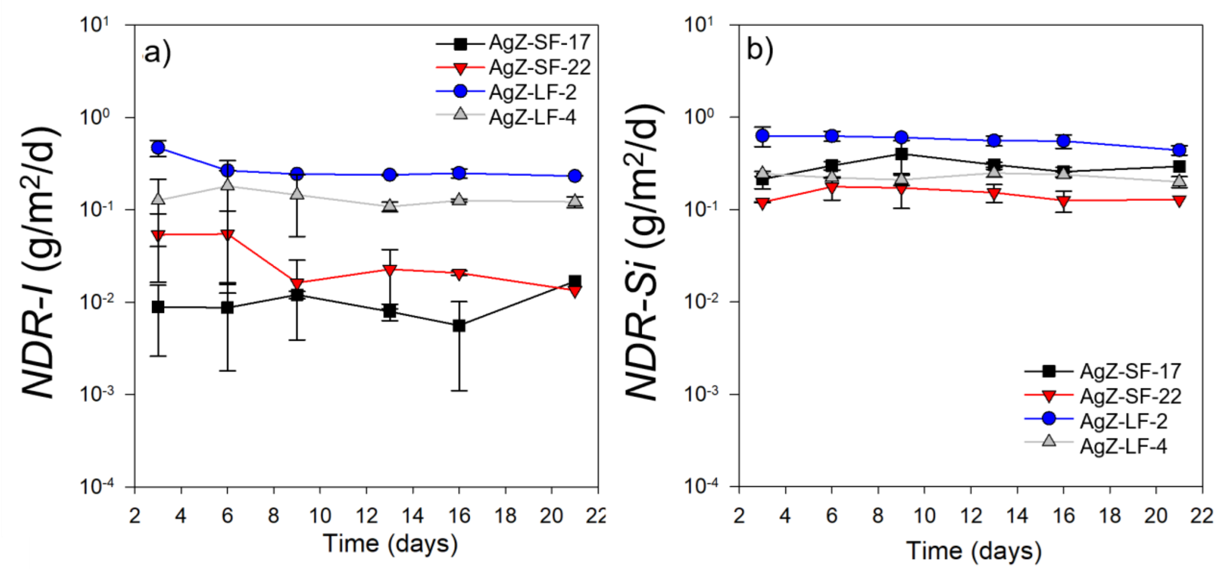

Figure S1. The (a) *NDR-I* and (b) *NDR-Si* measured following the Interval B sampling schedule for the AgZ samples. Error bars show the standard deviation from the average of two replicates.

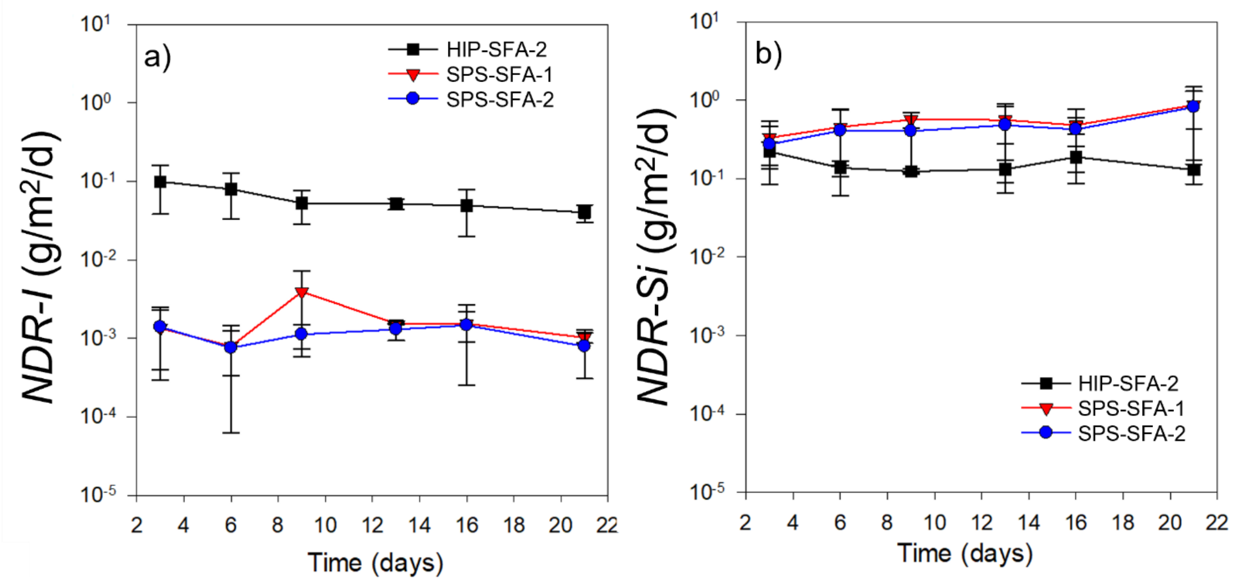

Figure S2. The (a) *NDR-I* and b) *NDR-Si* for the SFA samples. Error bars show the standard deviation from the average of two replicates.

S.5 SEM/EDS maps of AgZ-SF samples prior to testing

The following SEM micrographs and EDS maps show the differing microstructures of the AgZ-SF samples prior to performing semi-dynamic leach tests.

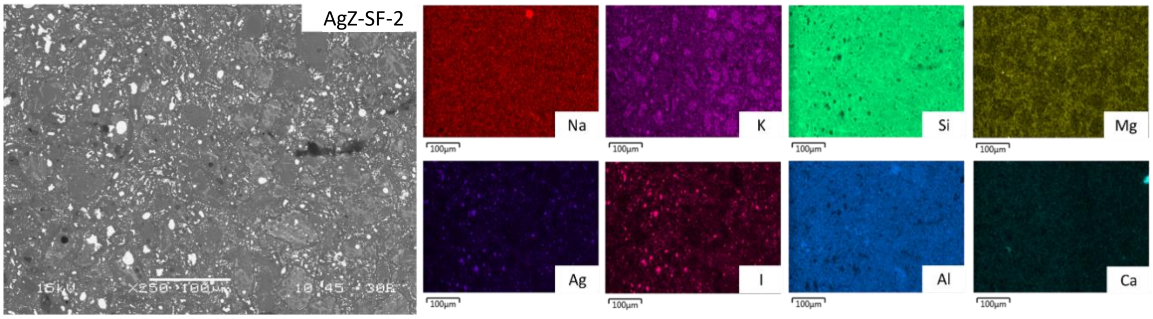

Figure S3. Comparison of the SEM micrograph and EDS maps of AgZ-SF-2 before testing.

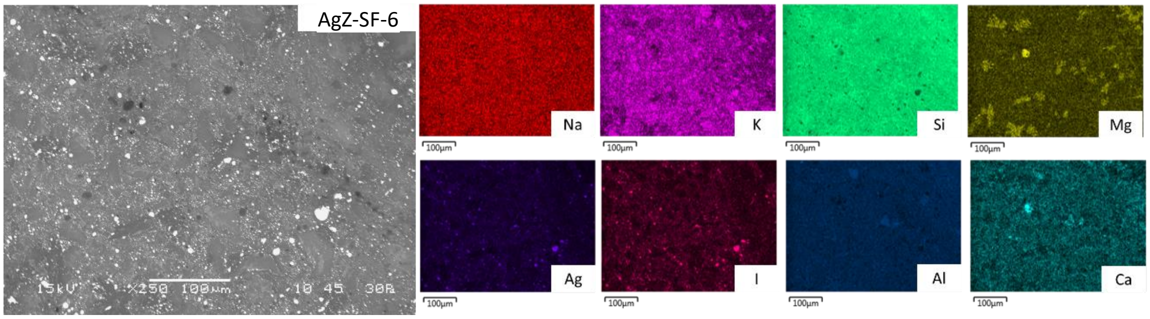

Figure S4. Comparison of the SEM micrograph and EDS maps of AgZ-SF-6 before testing.

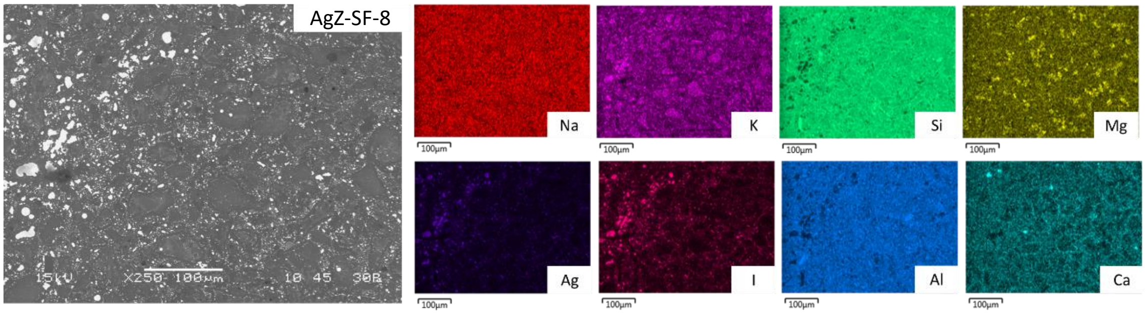

Figure S5. Comparison of the SEM micrograph and EDS maps of AgZ-SF-8 before testing.

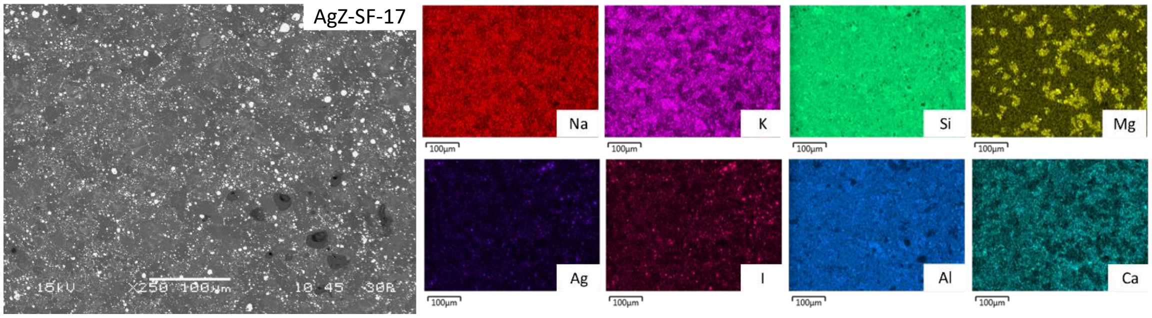

Figure S6. Comparison of the SEM micrograph and EDS maps of AgZ-SF-17 before testing.

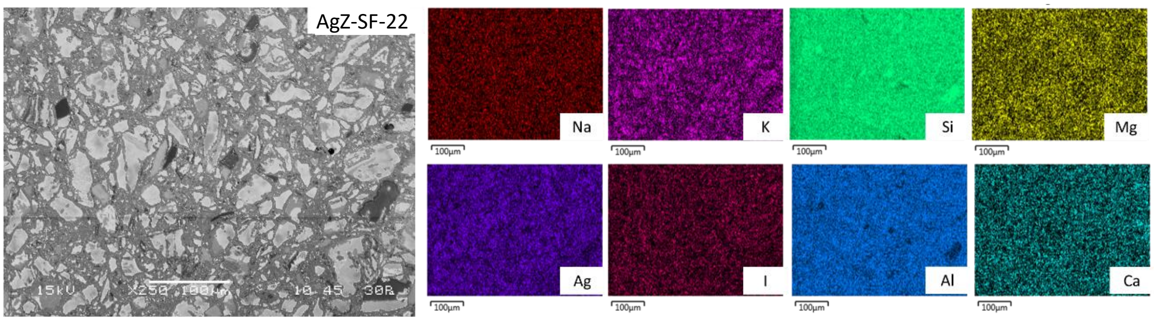

Figure S7. Comparison of the SEM micrograph and EDS maps of AgZ-SF-22 before testing.

S.6 SEM/EDS maps of AgZ-LF and SFA samples prior to testing

The following SEM micrographs and EDS maps show the differing microstructures of the AgZ-LF and SFA samples prior to testing. The green ring region (Figure S8a) appeared to be composed of larger isolations of the AgI host phase compared with the brown region (Figure S8b), which had a mix of large and fine-grain AgI host phase particles. The µXRD analysis of the AgZ-LF-1 and AgZ-LF-2 samples detected more Ag-metal and a small amount of hematite (Fe_2O_3, likely origin of the brown color) within the brown region compared with the exterior, which had mullite ($Al_6Si_2O_{13}$, from the mordenite) and a small amount of a cubic (Na,K)Cl phase. The XRD patterns can be found in Figure S11 and Figure S12.

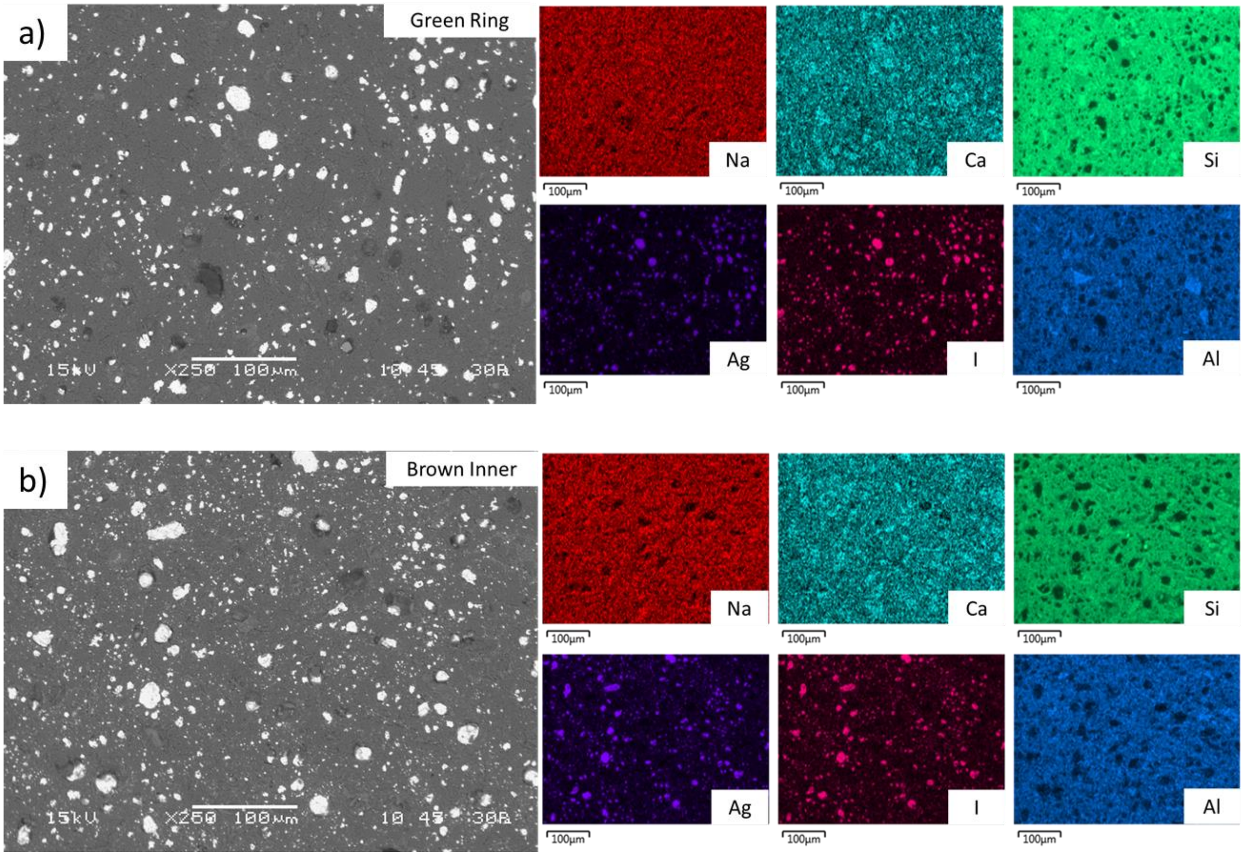

Figure S8. Comparison of the SEM micrograph and EDS maps of AgZ-LF-1 before testing for (a) the green outer ring and (b) the brown interior seen in the AgZ-LF-1 and AgZ-LF-2 AgZ samples.

The AgZ-LF-3 and AgZ-LF-4 samples had smaller but more evenly distributed AgI crystals. Within the green area (Figure S9a), the SEM micrographs show a large number of Ag isolations, with the AgI host phases being the larger particulates. The brown region (Figure S9b) contained veins of AgI host phase. This difference in distribution of the AgI host phase between the regions is evident in the lower magnification SEM micrograph (Figure S9c). From μXRD analyses, the only measurable difference between the two regions was the green areas having a small amount of Ag metal not associated with I (Figure S13, Figure S14). The Ag utilization clearly impacted the resulting waste form microstructure; however, the origin of this microstructure difference is unknown and will be a focus of future work. This study assessed differences in corrosion behaviors of these distinct HIP AgZ samples, allowing correlations to be drawn between sample processes, the resulting microstructure, and corrosion behavior.

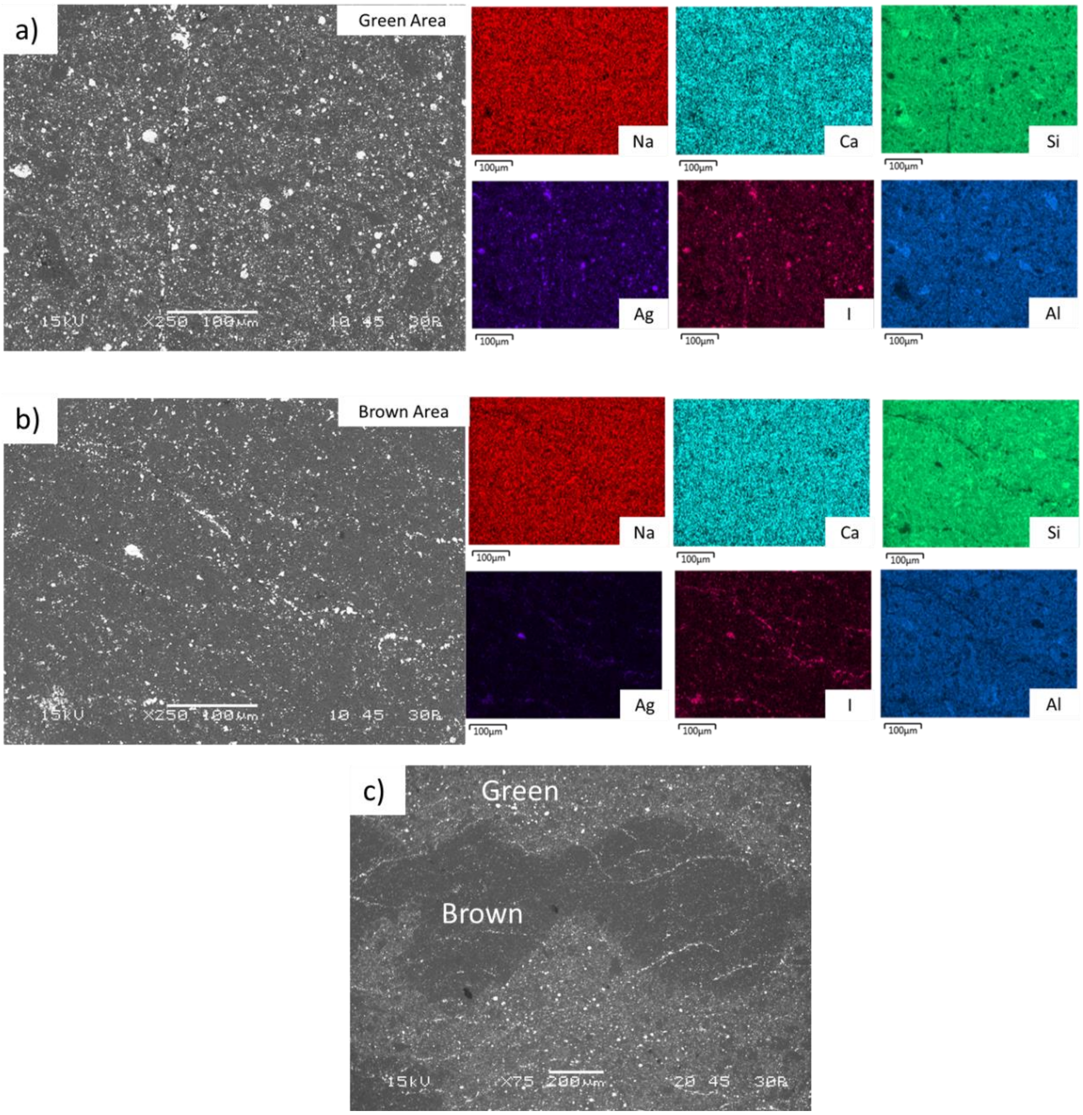

Figure S9. Comparison of the SEM micrograph and EDS maps of AgZ-LF-4 before testing for (a) the green outer ring and (b) the brown interior common in the AgZ-LF-3 and AgZ-LF-4 AgZ samples; (c) SEM micrograph at lower magnification showing the "green" and "brown" regions.

For the SFA samples processed with either HIP or SPS, distinctly different microstructures were observed (Figure S10). The HIP SFA sample had large veins of AgI host phase dispersed between Si-rich matrix phases (Figure S10a). The SPS SFA samples had islands of both Si and Ag (Figure S10b), with the AgI host phase spread along the particle boundaries and within the Ag-rich islands.

Discrete regions of S were also observed on both samples coming from the thiol linkages of the SFA.

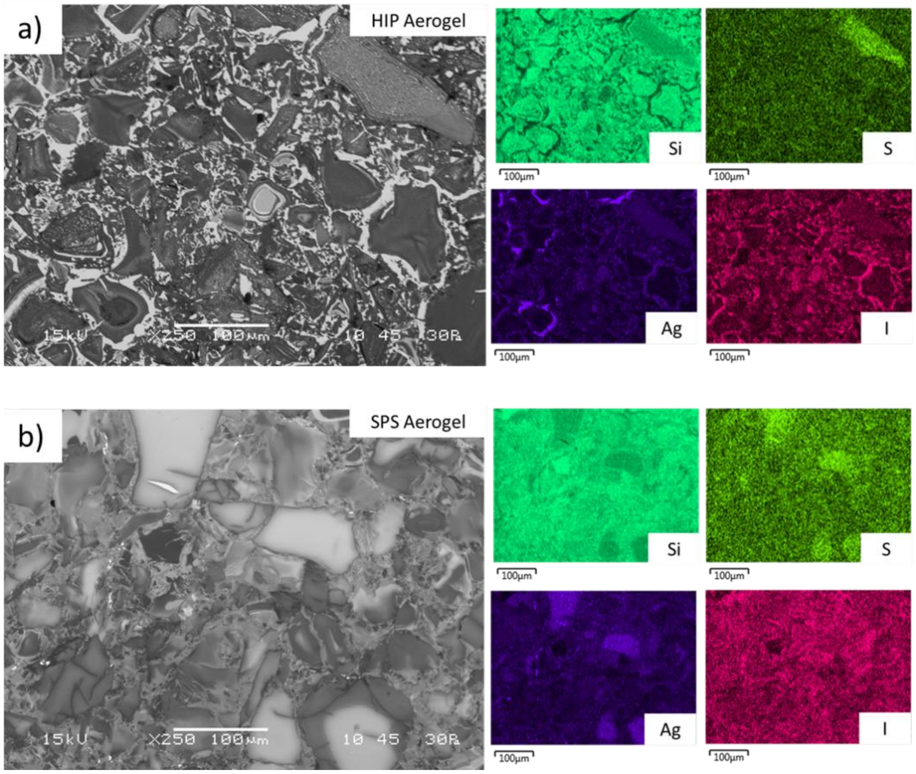

Figure S10. Comparison of the SEM micrograph and EDS maps before testing for (a) the HIP SFA samples and (b) the SPS SFA samples.

S.7 µXRD analysis of AgZ-LF Samples

The AgZ-LF-2 sample had dark and light regions, but they were less distinguishable and the spectra for both were very similar. As such, only the darker region spectrum is shown in Figure S13. Peak matches are shown in the top right corner. The light area was quartz, AgI, and a little mullite ($Al_6Si_2O_{13}$). There were a few very weak unidentified peaks. The darker area contained a tiny amount of Ag metal, the only significant difference between the two regions.

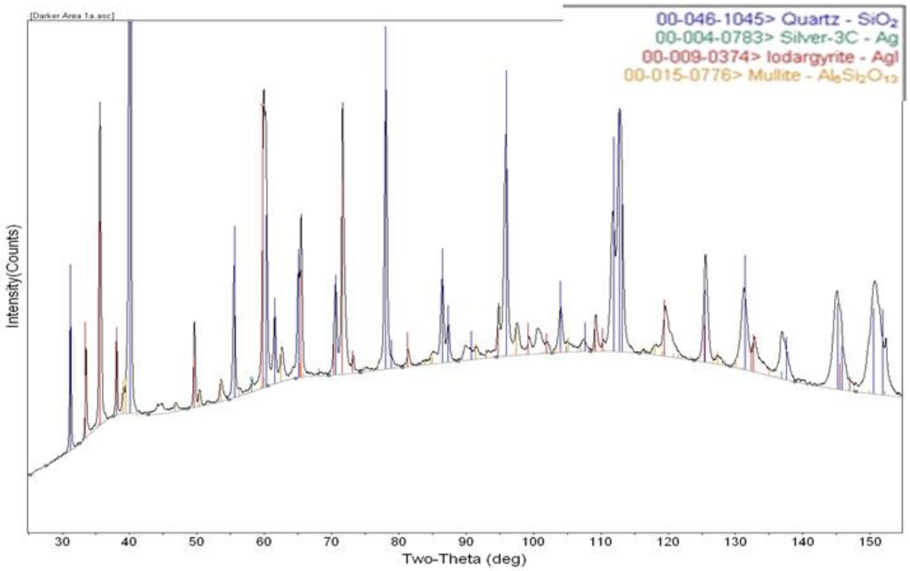

Figure S11. µXRD pattern for the AgZ-LF-2 sample (100% Ag use) with the peak matches in the upper right.

The AgZ-LF-2 sample was also characterized following the semi-dynamic leach test and the resulting pattern is shown in Figure S12. No changes were detected.

AgZ-LF-2

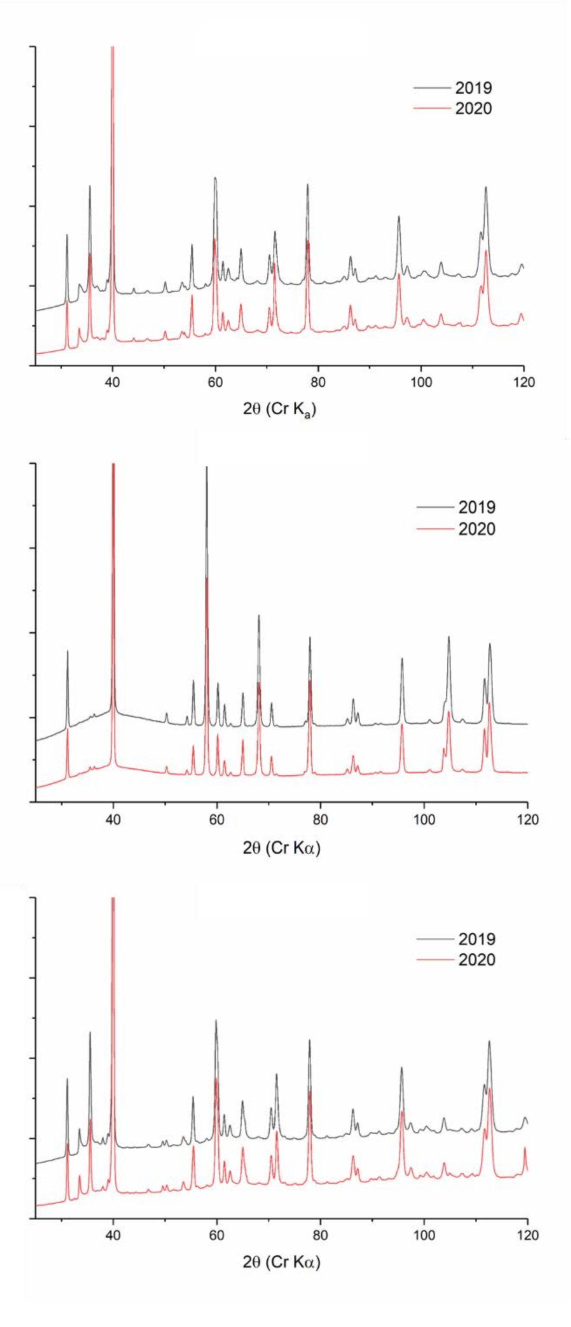

Figure S12. Pre-leach (black, 2019) and post- leach (red, 2020) μXRD patterns for AgZ-LF-2 (100% Ag use) on three regions of the sample surface.

The AgZ-LF-3 sample had both of its colored areas analyzed with μXRD, Figure S13. The lighter outer area (tan colored) contained a small amount of Ag metal along with a smaller quantity of a cubic compound [possibly (Na,K)Cl], quartz, AgI, and a small amount of mullite from the zeolite. The darker, inner regions in AgZ-LF-3 had much more Ag metal and a minor contribution from hematite. There was no mullite, and less of the cubic [perhaps (Na,K)Cl] compound.

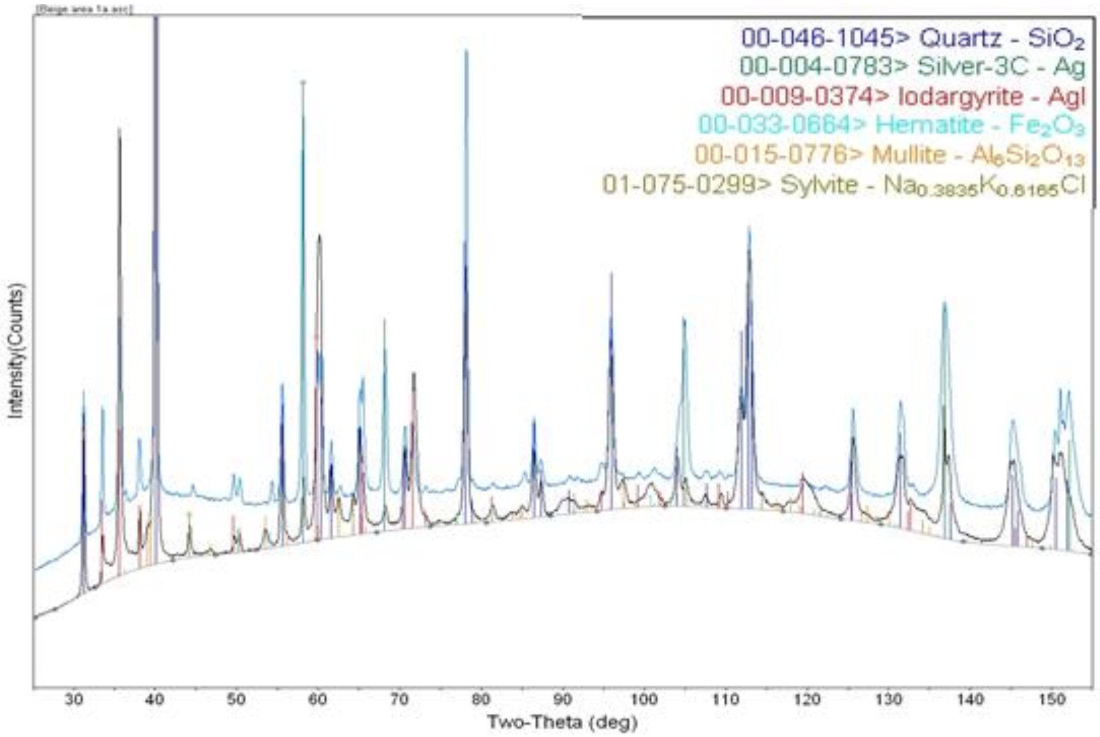

Figure S13. μXRD pattern for the AgZ-LF-3 sample (33% Ag use) with the pattern from the green region in black and from the brown region in light blue with peak matches in the upper right.

The AgZ-LF-3 sample was again characterized following the semi-dynamic leach test and the resulting patterns are shown in Figure S14. Little to no changes were detected before and after leaching, although Ag metal in one region was reduced, but the orange-region did not show this and contained the most Ag metal.

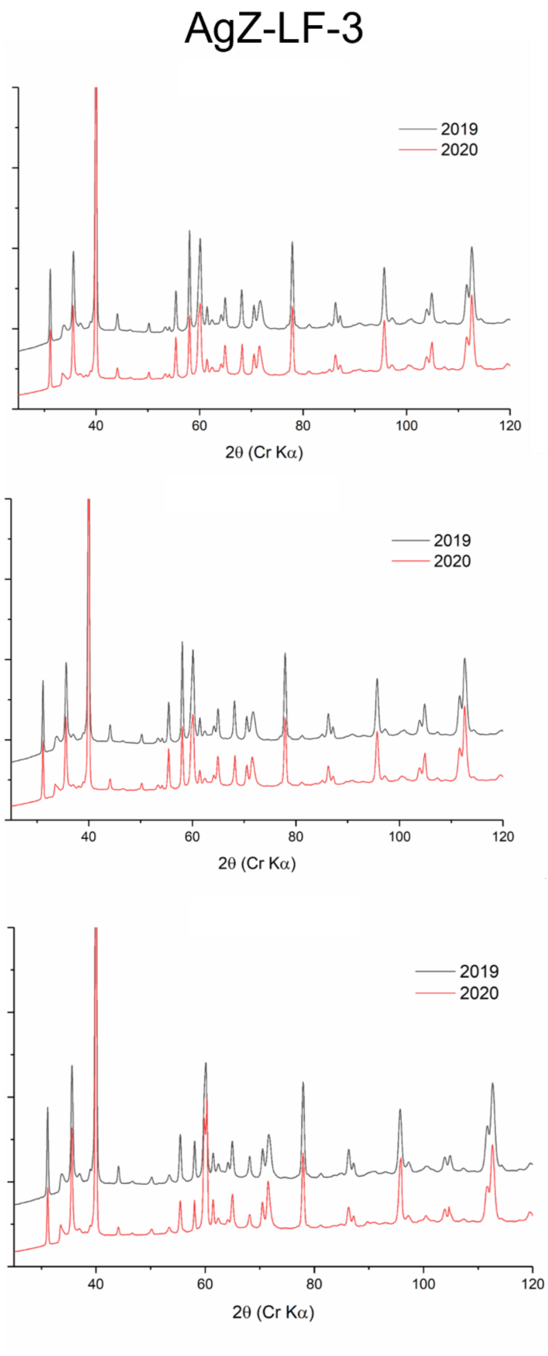

Figure S14. Pre-leach (black, 2019) and post- leach (red, 2020) μXRD patterns for AgZ-LF-3 (33% Ag use) on three regions of the sample surface.

S.8 XRD analysis of SFA Samples

XRD analysis (phase identification and Rietveld refinement) was conducted on one SPS SFA sample and on two regions (center and edge) of one HIP SFA sample. The microstructure composition was quantified with XRD in the SPS sample. Although the HIP sample was largely amorphous, AgI was still detected in the HIP sample at both the center and edge of the sample.

Table S5. Phase identification and Rietveld refinement of SPS-SFA and HIP-SFA samples.

Sample<br>R_{wp}		ICSD collection code	Chemical formula fraction (wt%)
SPS-SFA-1<br>4.7	Kalsilite	34350KAlSiO4	51
	Natrosilite	34688Na2Si2O5	40.2
	Silver	53759Ag	2
	Amorphous	-	6.8
HIP-SFA-1<br>center<br>5.7	Silver	Ag	1.5
	Miersite	56552γ-AgI	22.3
	Iodargyrite	21086-AgI	3.1
	Amorphous	-	73.1
HIP-SFA-1<br>edge<br>5.6	Silver	Ag	1.2
	Miersite	56552γ-AgI	9.4
	Iodargyrite	21086-AgI	2.1
	Amorphous	-	87.3

S.9 Using first samples from Interval B as a single test interval

In the design and development of an IWF, a rapid assessment of material durability would be beneficial to screen developmental stage samples. To evaluate the potential for using a single-interval 3-day leach test as rapid screening for IWF durability, the results of the first 3-day leach interval were compared to the results of the extended semi-dynamic leach test (Interval B).

Table S6 shows the *NDR-I* and *NDR-Si* from the first 3-day interval leach tests that were performed at 90°C on AgZ and SFA samples. The error was calculated as the standard deviation (±1σ) of the mean in duplicate runs where available. The *NDR-I* of AgZ-SF-22 was nearly an order of magnitude higher than the *NDR-I* of AgZ-SF-17, and the NDR-Si of the AgZ-SF-22 was marginally lower. The AgZ-SF-22 sample was dried at 450°C before being HIPed, and the processing time was 3 hours compared to the 12 hours used for preparing the non-dried AgZ-SF-17 sample. This suggests that a longer processing time and skipping the drying step prior to HIPing the sample could result in a more robust matrix and decrease the release of I.

For the large-form AgZ samples, AgZ-LF-2 had a higher iodine loading (100% Ag use) and had a larger *NDR-I* (0.47 ± 0.094 $g/m^2/d$) compared to AgZ-LF-4 (0.13 ± 0.087 $g/m^2/d$), which had lower iodine loading (33% Ag use). A similar trend was present for the *NDR-Si*, with the higher iodine-loaded samples having NDR-Si values of 0.63 ± 0.2 $g/m^2/d$ and the lower iodine-loaded samples having *NDR-Si* values of 0.24 ± 0.02 $g/m^2/d$. Duplicate measurements of the large-form HIPed AgZ showed only minor differences between the two tests, suggesting good reproducibility of the technique.

The SPS SFA samples (*NDR-I*: 0.0013 $g/m^2/d$, 0.0014 $g/m^2/d$) had over ten-fold lower *NDR-I* values than the HIP SFA sample (0.099 $g/m^2/d$). The *NDR-Si* values on the other hand all fell within the margin of error across the three SFA samples. This trend again shows that the SFA

samples have similar matrix durability but the SPS samples have improved host phase stability or isolation.

Table S6. *NDR* for iodine and silicon, for the various samples evaluated in the single sampling 3-day screening test.

IWF Type	Sample ID	***NDR-I*** **(g/m²/d)**	***NDR-Si*** **(g/m²/d)**
AgZ (small)	AgZ-SF-17	0.0089 ± 0.0063	0.21 ± 0.04
	AgZ-SF-22	0.054 ± 0.037	0.12 ± 0.0009
AgZ (large)	AgZ-LF-2	0.47 ± 0.094	0.63 ± 0.2
	AgZ-LF-4	0.13 ± 0.087	0.24 ± 0.02
SPS or HIP SFAs	SPS-SFA-1	0.0013 ± 0.00094	0.33 ± 0.2
	SPS-SFA-2	0.0014 ± 0.0011	0.27 ± 0.2
	HIP-SFA-2	0.099 ± 0.061	0.22 ± 0.07

When comparing data from the 3-day single-interval test, replicates on freshly polished samples should be performed, when possible, to provide a measure of error. A difference of greater than an order of magnitude in *NDR* values measured in a single 3-day interval test can be considered significant when used for screening samples of the same material class. For example, no difference in overall durability can be determined for the AgZ samples tested, while the HIP-SFA samples can be labeled as less durable than the SPS-SFA counterpart based on the *NDR-I*. The relative stabilities and trends across samples observed in the 3-day single interval test were consistent with the results of the extended leach test (Figure 4 of the main text).

S.10 Images of samples from tests conducted with different leachant pH values

Following the corrosion testing, the sample surfaces were imaged for any significant changes in the microstructure. At pH 7 (Figure S15) and pH 11 (Figure S16), little change was observed in the microstructures and the interfaces between the phases in the microstructure. At pH 4 (Figure S17), there appeared to be selective etching between the microstructure phases as seen in the AgZ-LF-1-rep1 and AgZ-LF-4-rep1 images. Further characterization is required to assess the extent of selective etching across pH regimes.

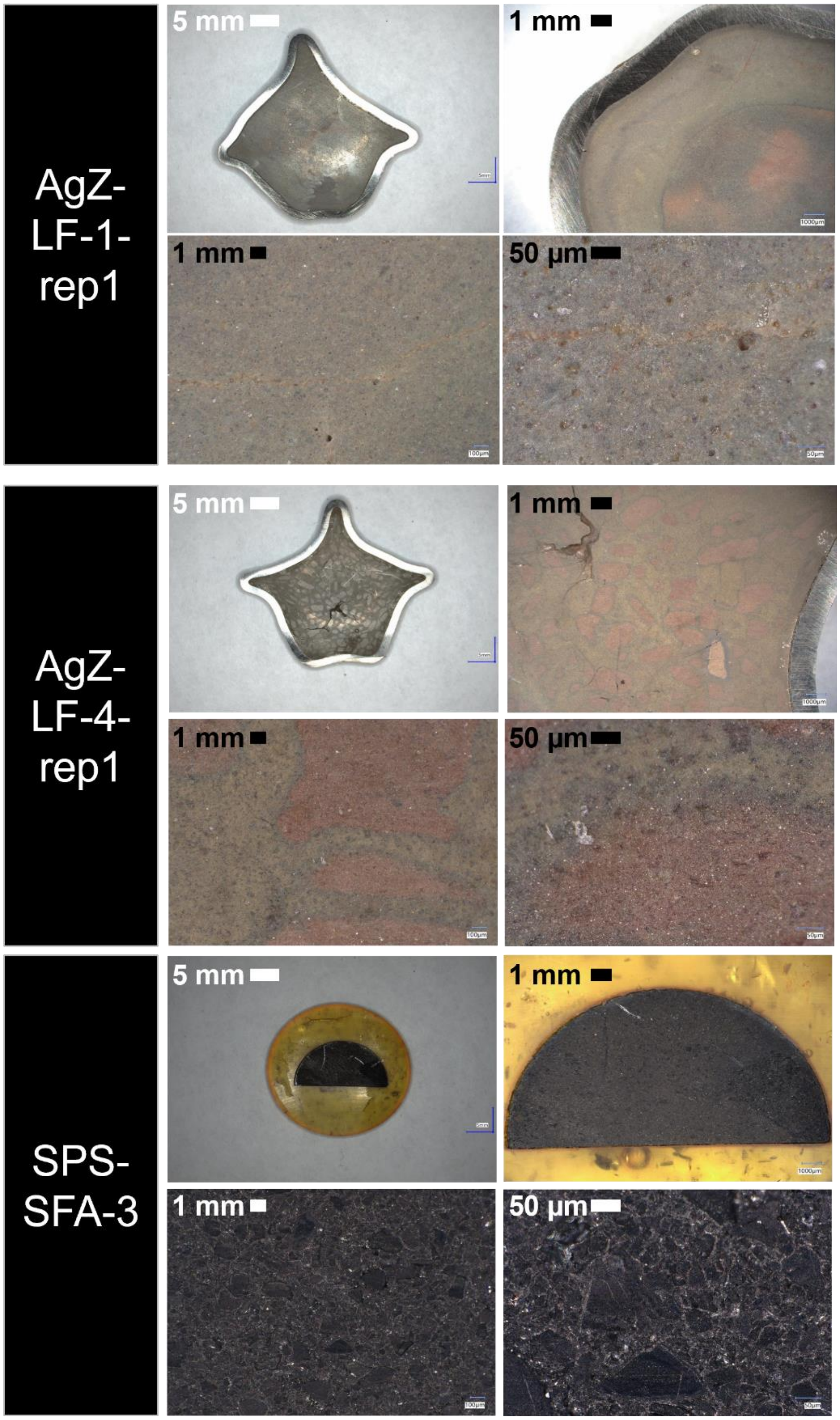

Figure S15. Example photographs of the samples following the 7-day (Interval C) test at pH 7 and 90 °C.

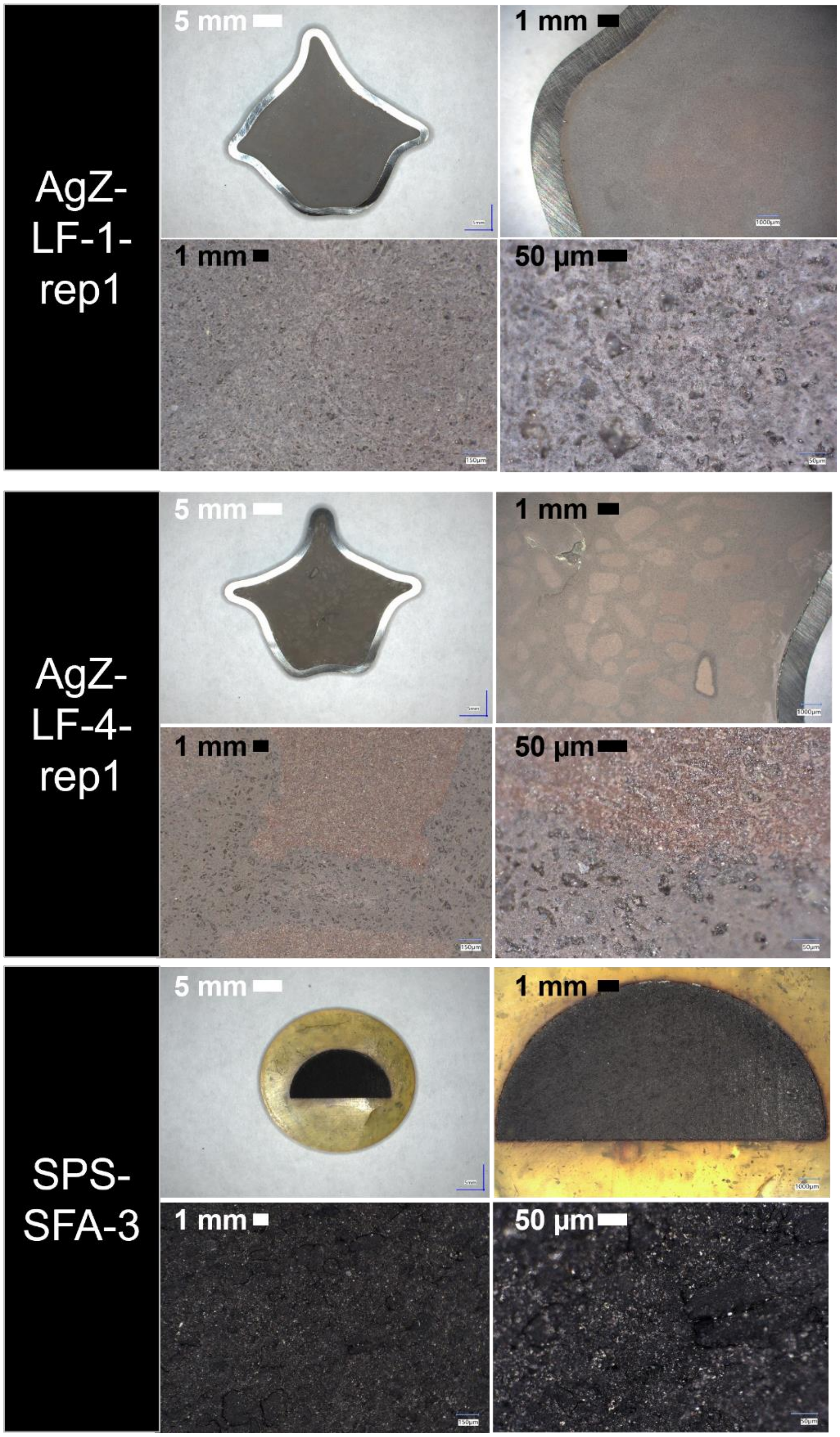

Figure S16. Example photographs of the samples following the 7-day (Interval C) test at pH 11 and 90 °C.

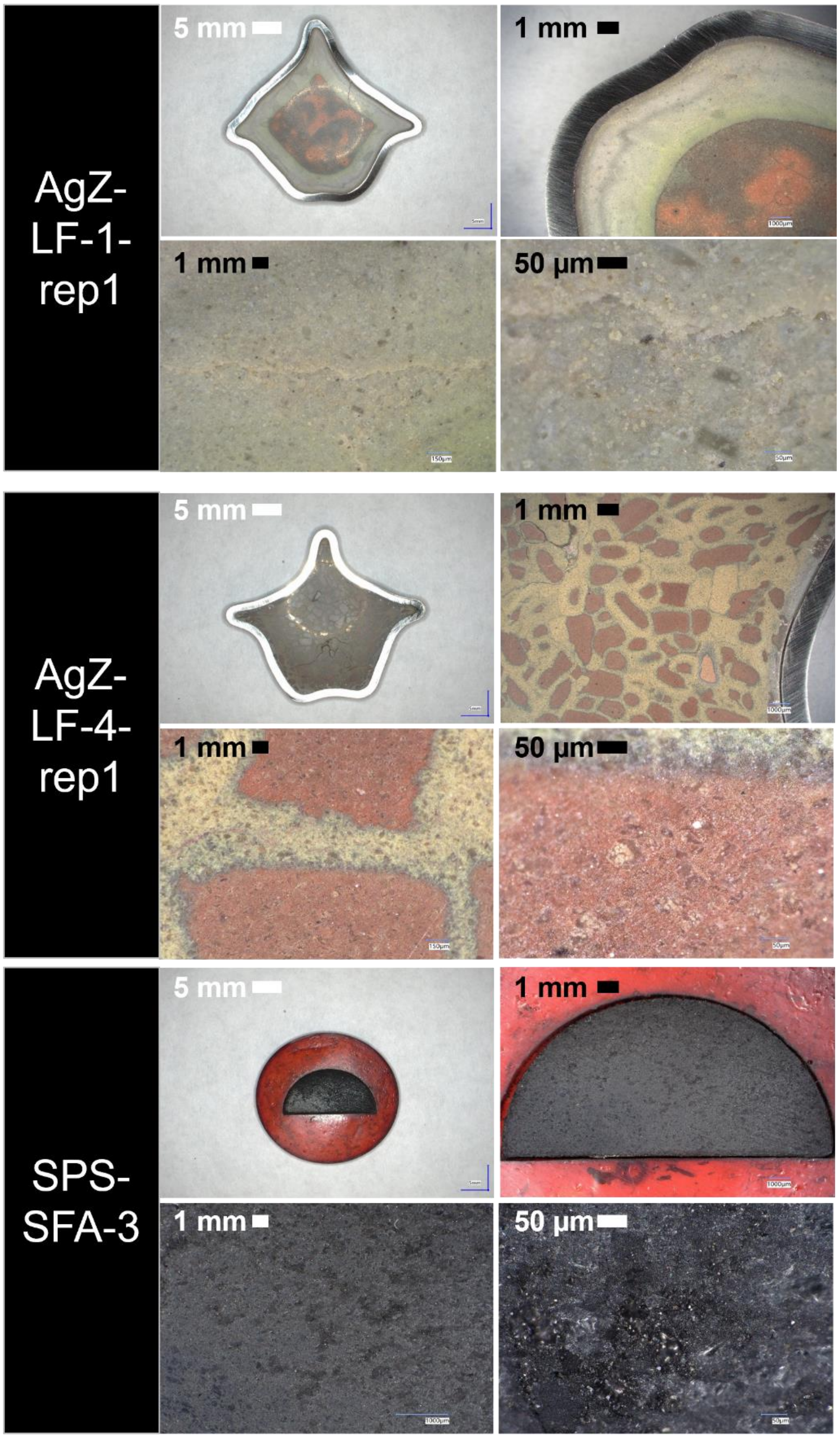

Figure S17. Example photographs of the samples following the 7-day (Interval C) test at pH 4 and 90 °C.

S.11 Effects of surface-area-to-volume ratio

While a standardized *S/V* ratio is suggested in the present study, the irregular size and shape of some IWF samples may require alternate *S/V* ratios to be used. An example scenario is larger-form samples (e.g., AgZ-LFs) with a single exposed face possibly being too thick for the required volume of leachant to cover the sample depending on the size of the test vessel. To assess whether varying the *S/V* ratio would change the dilution within a reactor, a set of leach tests were carried out at two *S/V* ratios for each sample. The measured *NDR-I* and *NDR-Si* from a 3-day test interval are shown in Table S7. Only minor changes in the *NDR-I* and *NDR-Si* were observed between the tests with different *S/V* ratios, in most cases < 3×. This observation indicates that changing the *S/V* ratios by <10× may not overly impact the *NDR* when comparing samples of the same material class. Therefore, it is suggested that an *S/V* ratio of 1 cm^{-1} be used where possible and where sample geometry allows to increase the likelihood of measurable dissolved constituents being present in the leachate.

Table S7. Normalized dissolution rates for I and Si at the 3-day sampling interval with variable *S/V* volume ratios. When duplicate experiments were performed, values are displayed as mean ± standard deviation.

Sample ID	Sample size (n)	Target *S/V* (cm^{-1})	Actual *S/V* (cm^{-1})	NDR-I ($g/m^2/d$)	NDR-Si ($g/m^2/d$)
**AgZ-LF-2**	1	0.1	0.09	0.14	0.23
	2	0.01	0.01 ± 0.00	0.47 ± 0.094	0.63 ±0.15
**AgZ-LF-4**	1	0.1	0.09	0.022	0.1
	2	0.01	0.01 ± 0.00	0.13 ± 0.087	0.24 ± 0.015
**SPS-SFA-2**	2	0.1	0.11 ± 0.01	0.0014 ± 0.0011	0.27 ± 0.19
	1	0.3	0.29	0.001	0.22
**HIP-SFA-2**	2	0.1	0.12 ± 0.03	0.099 ± 0.061	0.22 ± 0.074
	1	0.8	0.8	0.0018	0.13

S.12 Effects of masking

SPS and HIP SFA samples were used to assess the impacts of leaving the bottom face (which was in contact with the floor of the vessel) unmasked. The resulting *NDR-I* (Figure S18a) for the masked and unmasked samples were nearly identical to one another. However, some differences were observed between the *NDR-Si* for the masked and unmasked samples. For example, the *NDR-Si* for the masked HIP SFA sample was an order of magnitude higher than it was for the unmasked sample. To alleviate any possible contamination from uncured masks and discrepancies in analyses, it is suggested to not use a mask if the sample is flat or use epoxy resin that is stable at the test temperatures.

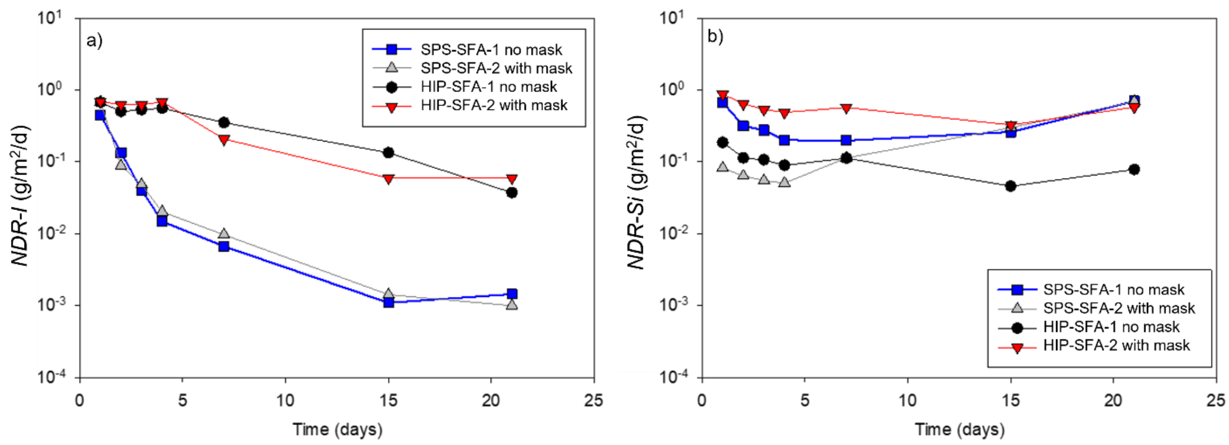

Figure S18. Comparison of the a) NDR-I and b) NDR-Si for SFA samples (SPS-SFA-1, SPS-SFA-2, HIP-SFA-1, HIP-SFA-2) tested with an RTV silicone mask on one surface of the sample and where no mask was present and the sample was placed at the bottom of the reactor. The leach test followed sample Interval A at 90°C.

S.13 Effects of surface finish

For mechanically durable samples, the test face should be polished to reduce potential variations in surface area from rough sample sectioning cuts or to refresh a sample used in a previous corrosion test. While a more-even surface is achieved with an increased degree of polishing, this process also removes material and could hinder the reuse of unique samples. To define the optimum polishing finish for IWF, leach tests were carried out on the AgZ-LF samples that were prepared with differing final polishing steps, and the results are shown in Figure S19. The samples were tested after polishing to one of the following finishes: colloidal silica (finest polish), 2400 grit silicon carbide (SiC), 1200 grit SiC, or 600 grit SiC. For AgZ-LF-1 and AgZ-LF-2 (which are replicate samples), the *NDR-I* (Figure S19a) and *NDR-Si* (Figure S19b) were similar with each polishing finish. There was a small exception in which the AgZ-LF-1 sample with a 1200 grit finish had a higher *NDR-I*. For the AgZ-LF-3 and AgZ-LF-4 replicate samples, the finer polished samples had slightly lower *NDR-I* (Figure S19c) values and similar *NDR-Si* (Figure S19d) values. The results of the surface finish testing suggest that a 600-grit polish can be used to minimize sample loss during repolishing for reuse of samples and to minimize dissolution of AgI.

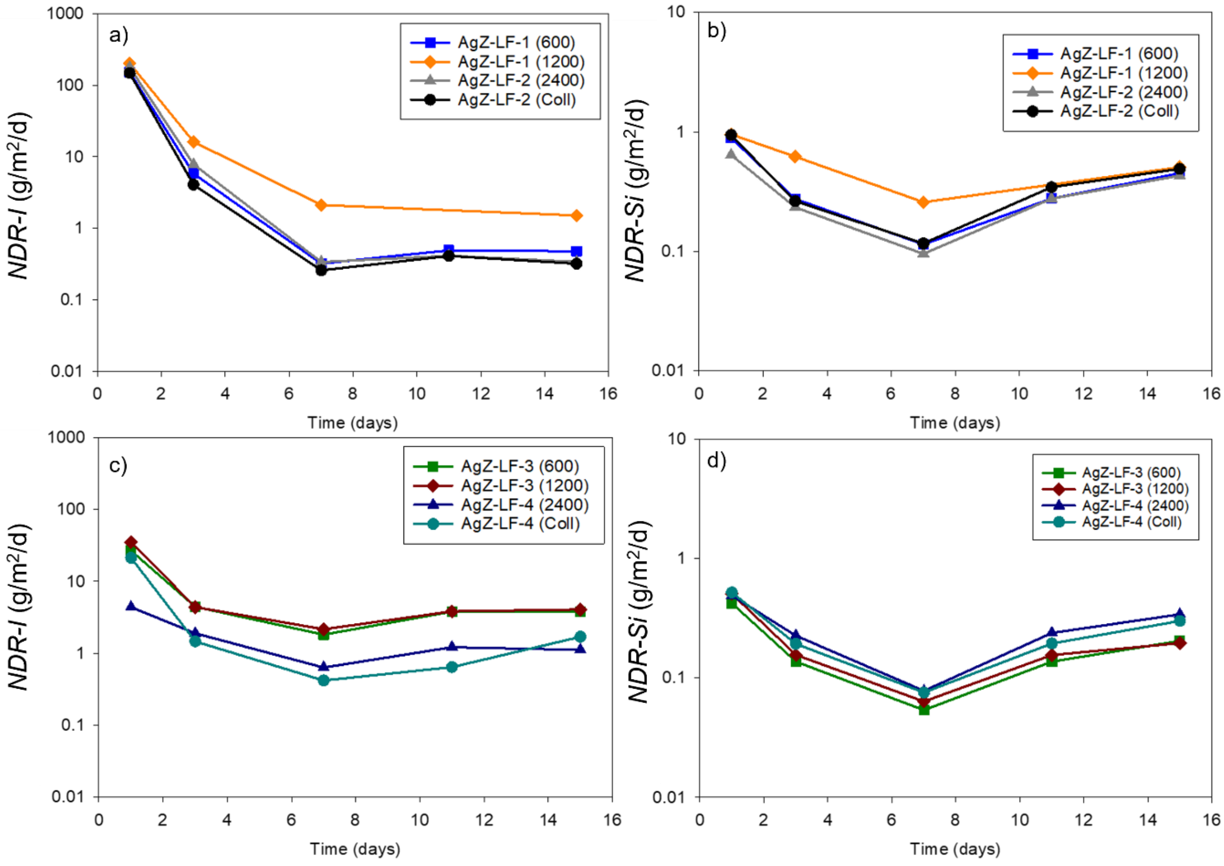

Figure S19. Comparison of the (a), (c) *NDR-I* and (b), (d) *NDR-Si* for the (a), (b) AgZ-LF samples and (c), (d) AgZ-SF samples prepared with different surface finishes ranging from 600 grit through colloidal silica (Coll, finest polish).

S.14 Post-corrosion images of AgZ-SF samples

A benefit of using monolithic samples is that it facilitates analysis of the corroded IWF surface following leach intervals or at the conclusion of the test to observe any microstructural changes. It is important to couple this microstructural information with the solution results to understand the controlling corrosion mechanism(s) of the IWF and ensure the mechanism(s) is sufficiently represented in a CCRM. Analysis of the solid sample surface also distinguishes the behavior of the individual phases as solution-based rates are a combination of release from all phases.

SEM images were collected both before and after semi-dynamic leach tests to observe any evolution of the sample microstructure. The same area on the sample surface was imaged when possible. If the original area could not be imaged after testing the interpretation was more subjective. Samples AgZ-SF-2, AgZ-SF-17, and AgZ-SF-22 showed little to no change in their microstructures following testing (Figure S20, Figure S21, Figure S22). Sample AgZ-SF-6 showed a newly exposed host phase region of AgI (denoted by a white arrow in Figure S23b). A similar behavior was observed on the AgZ-SF-8 sample shown in Figure S23g, where a cluster of the AgI host phase expanded following testing. Both samples were HIPed at 900°C and displayed high initial *NDR-I*, suggesting that the high HIP temperature can destabilize the AgI host phase in the small-scale HIP samples or produce free iodine available for release. Since the release is immediate upon exposure, this may indicate that the AgI host phase initially exposed in the 900°C samples may be less stable or have free iodine present than that in the lower-temperature HIP samples. Sample AgZ-SF-18, which was HIPed at the lowest temperature (525°C), displayed the most extensive matrix phase corrosion of the AgZ-SF samples, as seen in Figure S23j. Many of the large Si-rich regions have lost their definition and more AgI host phase has been exposed. AgZ-SF-18

had the highest NDR-Si (Figure 2 of the main text), and the post-corrosion analysis confirmed attack of the matrix phase.

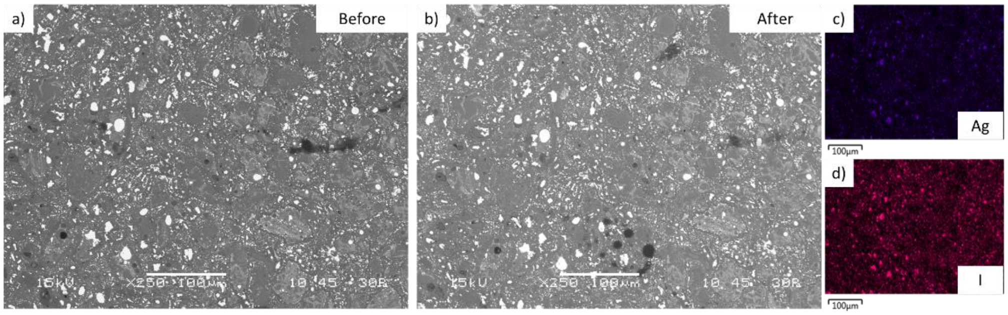

Figure S20. SEM Comparison of the microstructure of the AgZ-SF-2 sample a) before and b) after testing and the corresponding EDS maps for c) Ag and d) I from the corroded sample.

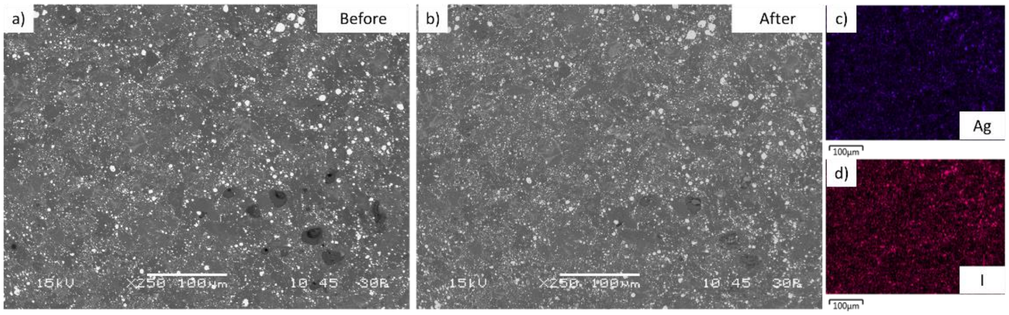

Figure S21. SEM Comparison of the microstructure of the AgZ-SF-17 sample a) before and b) after testing and the corresponding EDS maps for c) Ag and d) I from the corroded sample.

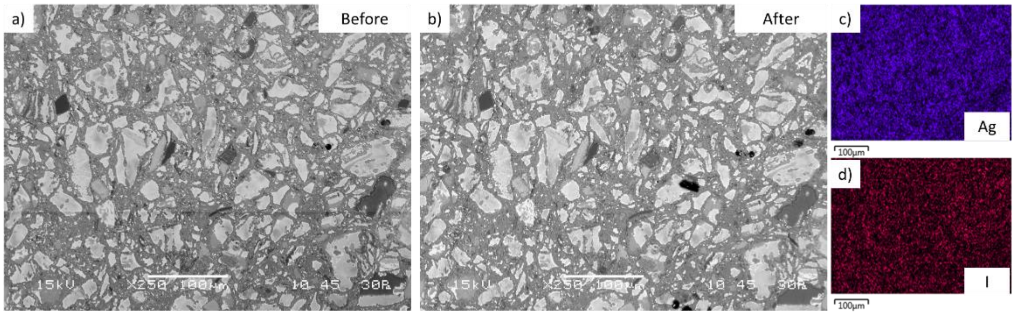

Figure S22. SEM Comparison of the microstructure of the AgZ-SF-22 sample a) before and b) after testing and the corresponding EDS maps for c) Ag and d) I from the corroded sample.

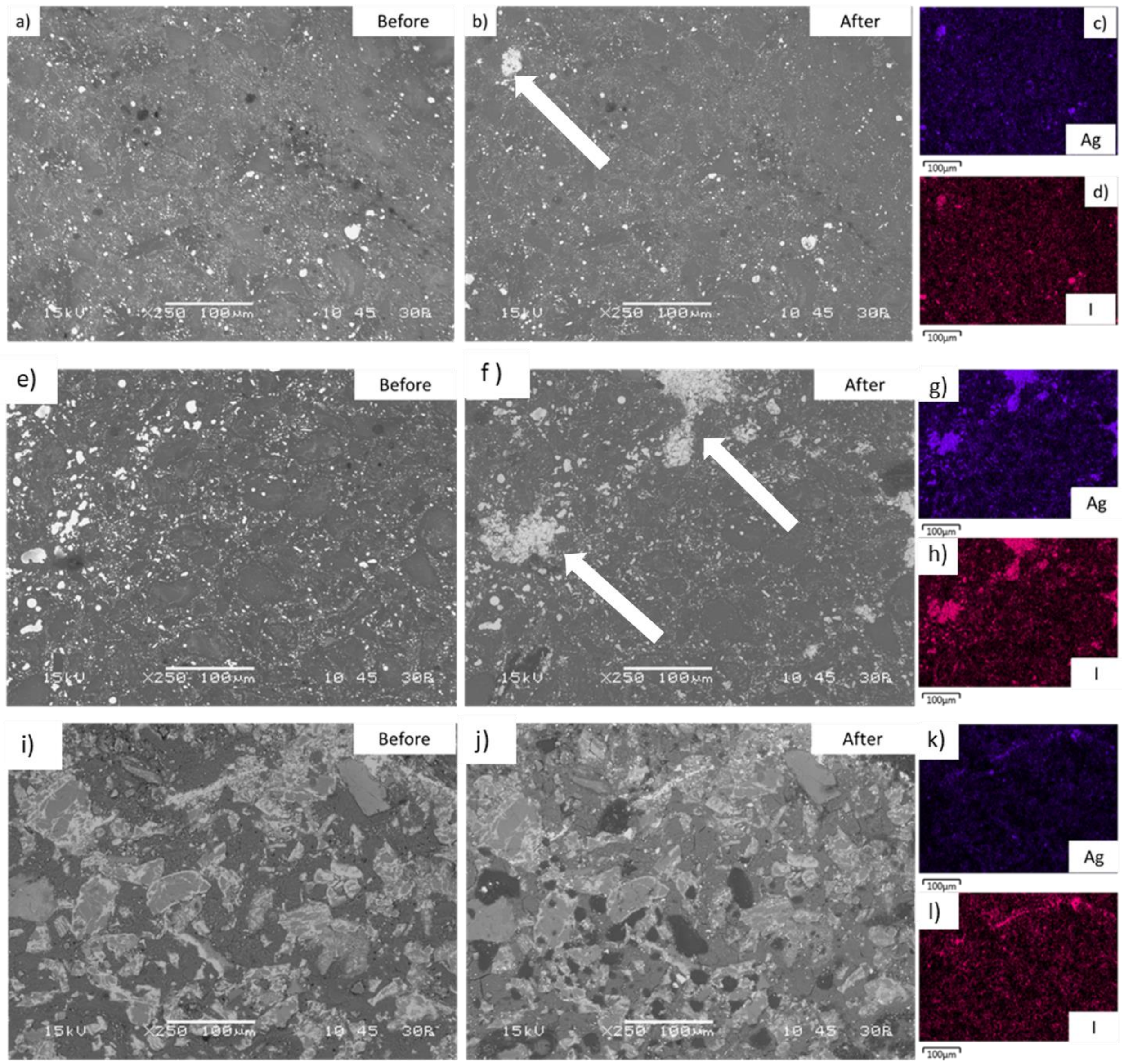

Figure S23. Comparison of the microstructure of the AgZ-SF-6 sample (a) before and (b) after testing as well as the corresponding EDS maps for (c) Ag and (d) I from the corroded sample. The white arrow highlights an exposed host phase following testing. The same is shown in (e)-(h) for AgZ-SF-8 and in (i)-(l) for AgZ-SF-18. The same location of the sample shown in the left column is shown in the right column.

The large-form (AgZ-LF) samples also displayed microstructural evolution following testing that was consistent based on the "color" of the area. Due to the large sample size, the same area on the surface was unable to be located for the post-corrosion testing. The green regions, seen

in the outer ring of AgZ-LF-1 and AgZ-LF-2 and distributed throughout the AgZ-LF-3 and AgZ-LF-4 samples, were corroded to expose more of the AgI host phase (Figure S24). Figure S24b shows the post-corrosion surface of the brown region in AgZ-LF-2. Within the green region, there was less evidence of change following corrosion (Figure S24g) for the AgZ-LF-3 sample. The AgZ-LF samples were HIPed at 900°C but did not display the large areas of host phase exposed following corrosion like the other small-form AgZ samples prepared at 900°C. This observation may have been the result of differing temperature profiles between the small-form and large-form samples during the HIP process. The observation also suggested matrix phases in the green regions are more susceptible to corrosion, but further analysis is required. There was little change in the mineralogical makeup of the regions after corrosion based on μXRD analyses.

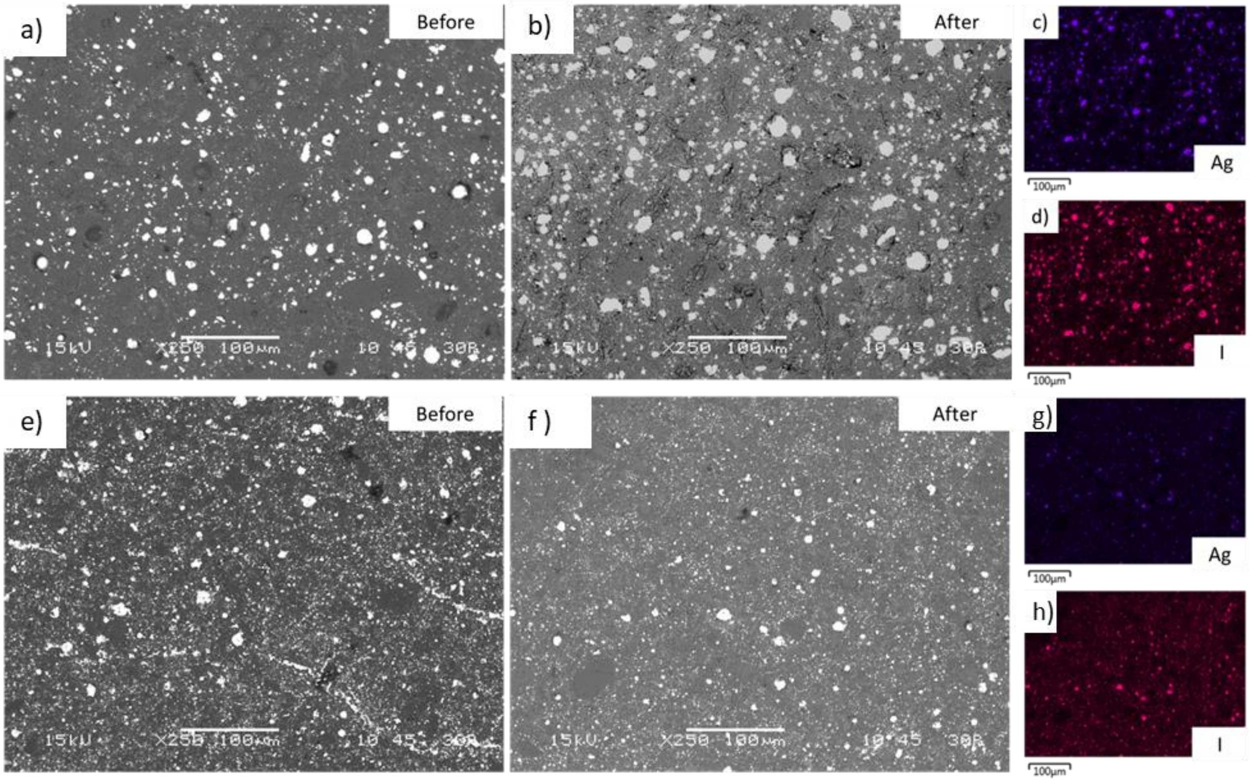

Figure S24. Comparison of the microstructure of the brown region from the AgZ-LF-2 sample (a) before and (b) after testing as well as the corresponding EDS maps for (c) Ag and (d) I from the corroded sample. The two areas shown in the SEM images are not the same. The same is shown in (e)-(h) for AgZ-LF-3.

The SPS SFA samples had much lower *NDR-I* values than their HIP SFA counterparts. Post-corrosion analysis of the HIP SFA sample (Figure S25a-d) showed a loss of the AgI from the veins running between grains of the silica matrix phases. However, the matrix phases themselves showed minimal change. Compared to the SPS SFA sample (Figure S25 e-h), the HIP sample's AgI host phase changed extensively following corrosion. Within the SPS SFA, some additional AgI host phase was exposed (highlighted by a white arrow in Figure S25f). Minimal changes to the matrix phases were observed on the SPS SFA surface. This observation aligns with the behavior shown in Figure 4 of the main text, where the HIP SFA has a higher *NDR-I* from the dissolving AgI host phase and a similar *NDR-Si* to the SPS SFA due to the matrix durability.

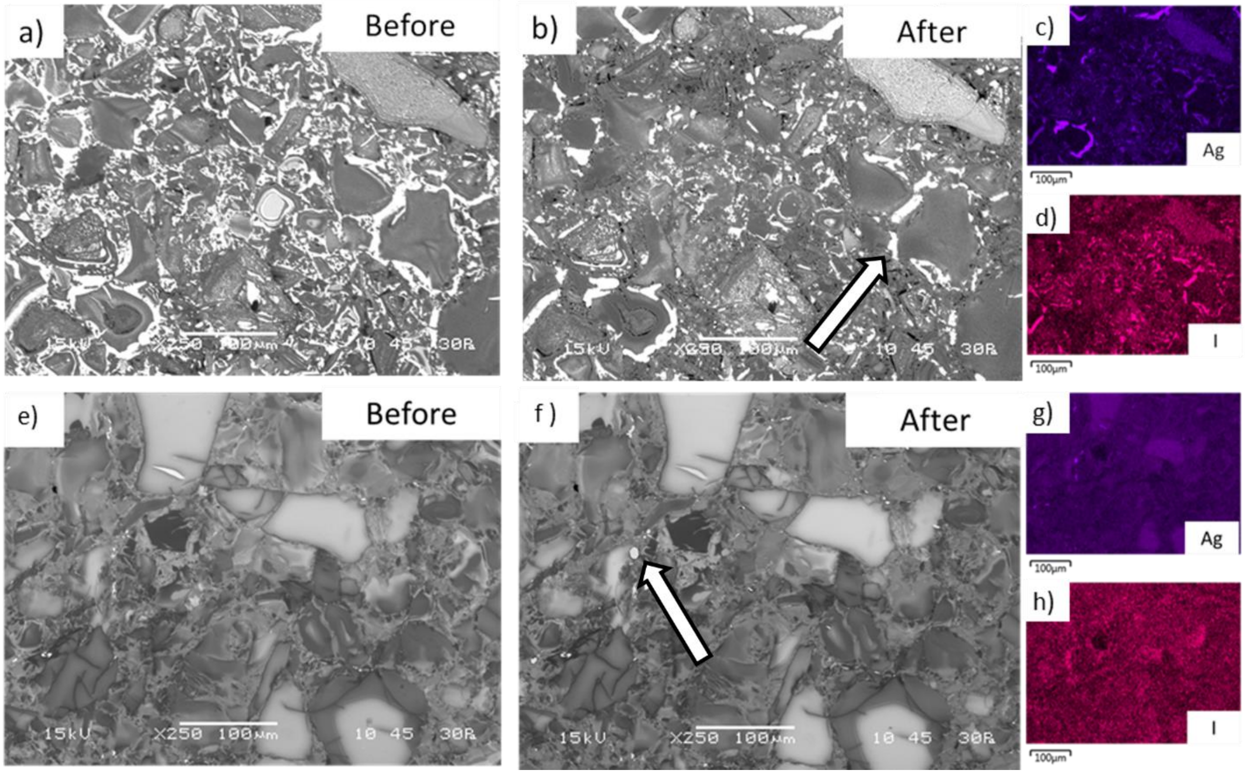

Figure S25. Comparison of the microstructure of the HIP SFA sample (a) before and (b) after testing as well as the corresponding EDS maps for (c) Ag and (d) I from the corroded sample. The white arrow highlights region of host phase that was lost in the testing. The same is shown in (e)-(h) for the SPS SFA sample, with the white arrow highlighting a host phase particle exposed following testing.
